# Characterisation of APOBEC3B-Mediated RNA editing in breast cancer cells reveals regulatory roles of NEAT1 and MALAT1 lncRNAs

**DOI:** 10.1038/s41388-024-03171-5

**Published:** 2024-09-25

**Authors:** Chi Zhang, Yu-Jing Lu, Mei Wang, Bingjie Chen, Feifei Xiong, Costas Mitsopoulos, Olivia Rossanese, Xiuling Li, Paul A. Clarke

**Affiliations:** 1 Centre for Cancer Drug Discovery, The Institute of Cancer Research, London, UK; 2 Shanghai Institute of Biological Products, Shanghai, China; 3 Guangdong Medicine-Engineering Interdisciplinary Technology Research Centre, School of Biomedical and Pharmaceutical Sciences, Guangdong University of Technology, Guangzhou, China; 4 GMU-GIBH Joint School of Life Sciences, Guangzhou Medical University, Guangzhou, Guangdong China

**Keywords:** Breast cancer, Mechanisms of disease, Oncogenes, Sequencing, Mutation

## Abstract

RNA editing is a crucial post-transcriptional process that influences gene expression and increases the diversity of the proteome as a result of amino acid substitution. Recently, the APOBEC3 family has emerged as a significant player in this mechanism, with APOBEC3A (A3A) having prominent roles in base editing during immune and stress responses. APOBEC3B (A3B), another family member, has gained attention for its potential role in generating genomic DNA mutations in breast cancer. In this study, we coupled an inducible expression cell model with a novel methodology for identifying differential variants in RNA (DVRs) to map A3B-mediated RNA editing sites in a breast cancer cell model. Our findings indicate that A3B engages in selective RNA editing including targeting NEAT1 and MALAT1 long non-coding RNAs that are often highly expressed in tumour cells. Notably, the binding of these RNAs sequesters A3B and suppresses global A3B activity against RNA and DNA. Release of A3B from NEAT1/MALAT1 resulted in increased A3B activity at the expense of A3A activity suggesting a regulatory feedback loop between the two family members. This research substantially advances our understanding of A3B’s role in RNA editing, its mechanistic underpinnings, and its potential relevance in the pathogenesis of breast cancer.

## Introduction

RNA editing is a dynamic post-transcriptional process that modulates transcript sequences without altering the underlying genomic DNA sequence [1]. It plays a crucial role in expanding proteomic diversity and regulating gene expression in higher eukaryotes. Among the diverse mechanisms of RNA editing, two major types of modifications have been identified: deamination of adenine to inosine (A > I) or cytidine to uracil (C > U). These modifications impact on the cellular proteome as they are read as guanosine and uridine respectively during translation, thereby altering protein sequences encoded by genomic DNA [2, 3].

In mammals, the APOBEC (apolipoprotein B mRNA-editing enzyme, catalytic polypeptide-like) protein family, alongside the activation-induced cytidine deaminase (AICDA), are prominent members of the cytidine deaminases known for their roles in genomic editing and immune response regulation. [4]. While AICDA is recognised for its role in C > U mutation of DNA during antibody gene diversification, the APOBEC proteins have gained attention for their broader-spectrum roles in RNA editing [3, 5]. The human APOBEC family comprises ten members, APOBEC1, APOBEC2, and APOBEC4, that have restricted tissue expression, and the APOBEC3 genes that are more widely expressed, though predominantly in immune cells [6]. Initially characterised for their ability to inhibit retroviruses and transposable elements by deaminating cytidines in single-stranded DNA, recent studies have revealed the involvement of APOBEC3 family members in RNA editing. Physiological conditions, such as interferon stimulation, hypoxia, and cellular crowding, can induce endogenous APOBEC3A (A3A)-mediated C > U RNA editing in monocytes and immune cells [7–9]. The induction of RNA editing by A3A under these circumstances suggests its involvement in cellular stress responses and immune modulation. Furthermore, A3A-mediated RNA editing of specific transcripts has been observed, indicating its potential impact on gene expression regulation and protein diversity [9, 10]. However, the prevalence and significance of other APOBEC3-mediated RNA editing in various biological contexts, including cancer is less well understood and remains an area of active investigation [3].

The last 10 years have witnessed the growing interest in the function of another member of the APOBEC3 family in cancer. APOBEC3B (A3B) has been implicated in driving genetic heterogeneity in cancers by inducing C > T transitions and C > G transversions at 5’-TCW motifs (W = A or T) [11, 12], a mutation pattern observed in more than 50% of primary breast cancers [11, 12]. Being the sole constitutively nuclear-localised member of the APOBEC3 family, A3B has emerged as one of the primary sources underlying the genomic APOBEC3 mutational signature identified in breast cancer [11, 13]. The discovery that several members of the APOBEC3 family exhibit RNA-binding capabilities has also fuelled speculation about a role for A3B in RNA editing [14]. This is supported by the findings that the catalytic C-terminal domain (CTD) of A3B shares a high degree of similarity to A3A that binds RNA with high affinity [15]. However, the identification of transcriptome-wide A3B-mediated RNA editing sites and their cellular or mechanistic consequences in normal tissue or cancer cells remains unexplored. The challenges are twofold: firstly, even minimal genomic editing by A3B can be erroneously attributed to RNA editing due to the intricacies of the transcription process; secondly, RNA editing activities stemming from other members of the APOBEC3 family can confound the precise identification of A3B-specific RNA editing sites. Consequently, there is an imperative need for a more rigorous methodology to surmount these obstacles.

Here we set out to comprehensively map the RNA sites edited by A3B using a breast cancer cell model with inducible A3B expression in conjunction with a newly-developed methodology to identify differential variants in RNA (DVRs). By further analysis of these sites, we sought to understand the mechanism and functional impact underlying the RNA-editing function of A3B and the transcriptomic hotspots of A3B-editing.

## Results

### Identification of A3B-mediated RNA editing sites

**Fig. 1:**
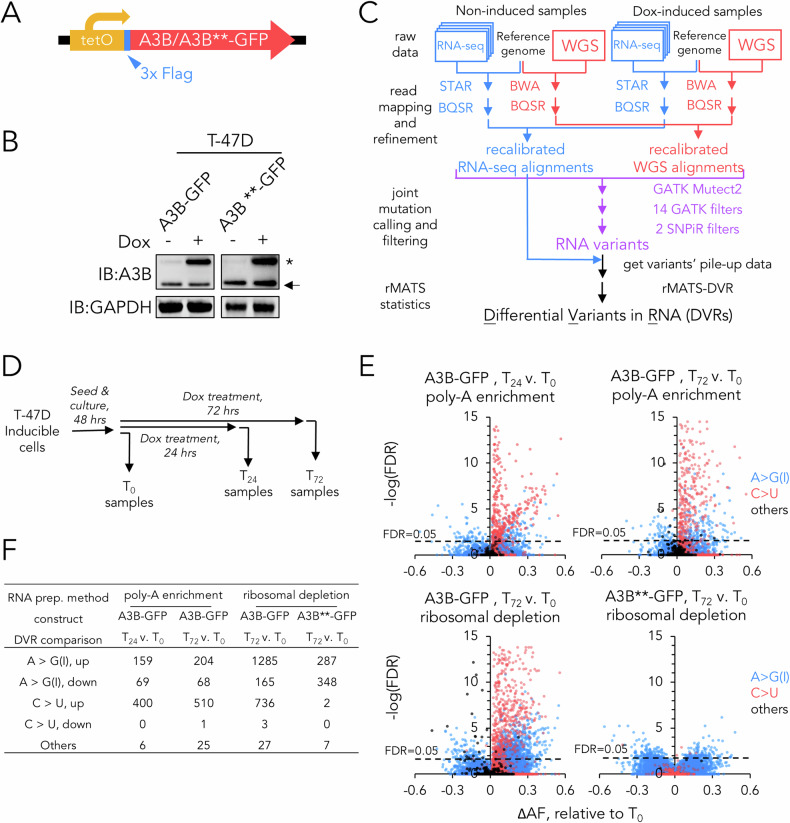
Identification of A3B-mediated DVRs using a lentiviral inducible system. **A** Schematic of the A3B wildtype or catalytically dead (**) lentiviral inducible expression cassette used in this study. **B** Immunoblots showing the expression of endogenous (arrow) and inducible-exogenous (asterisk) A3Bs following induction of T-47D cells using 100 ng/ml doxycycline for 48 h. GAPDH was used as loading control. **C** Schematic of the analysis pipeline for identifying A3B-mediated DVRs. For RNA-seq, *n* = 4 biological repeats were used. For WGS, *n* = 1 was used. **D** Schematic of treatment processes for RNA-seq samples. **E** Volcano plot depicting fraction change of alternative allele for each DVRs (ΔAF) upon doxycycline induction against FDR derived from likelihood-ratio test, where AF = (read depth of alternative allele)/(total read depth), and ΔAF = AF_dox-treated_-AF_dox-untreated_. In this study, FDR of ≤0.05 (dotted line) was applied as criteria for DVRs qualification. **F** Table showing break down of various types of DVRs in different sample groups relative to non-induced samples.

To discern RNA editing sites resulting from A3B activity, we constructed a lentiviral expression system enabling the doxycycline-induced expression of a Flag-tagged A3B fusion protein (Fig. 1A). By comparing A3B-induced to un-induced cells, we could identify RNA editing sites solely attributable to the elevated A3B catalytic activity. To mitigate potential interference from other APOBEC3 family members, notably A3A and A3G, our lentiviral inducible system was established in human T-47D breast cancer cells. T-47D cells are characterised by lower expression of A3A and A3G proteins relative to many breast cancer cell lines while having a moderate baseline expression level of A3B [16], which for our experiments ensured a rich endogenous interaction context relevant to the biological role of A3B. Furthermore, they possess a loss-of-function mutation in the *TP53* gene, making them resistant to programmed cell death triggered by A3B induction [17]. We meticulously regulated the degree of induced expression of the Flag-tagged A3B protein (Figs. 1B, S1A) to ensure its levels were akin to the peak levels observed for endogenous A3B in a range of breast cancer cell lines, as previously documented [11]. For control purposes, we also constructed an inducible system for an enzymatically inactive A3B variant (E65Q/E225Q), where both the N-terminal domain (NTD) and CTD catalytic domains are rendered inactive by mutation, henceforth referred to as A3B** [18]. This approach ensures the identification of A3B RNA editing sites that are physiologically pertinent within a breast cancer framework.

To identify A3B-editing sites in the T-47D transcriptome and distinguish the RNA sites from edited sites resulting from A3B-catalysed modification of genomic DNA, we used next-generation sequencing and an analysis workflow that was devised to identify the DVRs attributed to the induced A3B. This method joins the most salient features from the previously established VaDiR [19] and rMATS-DVR [20] workflows where: (i) False-positive RNA variants, arising from transcription of DNA’s single nucleotide variants (SNV), are filtered using GATK Mutect2 [21], which utilises joint mutation calling from pairing RNA sequencing (RNA-seq) with control whole genome sequencing (WGS) data; and (ii) The rMATS statistical approach which is employed on sample groups with multiple independent replicates (*n* = 4) to derive highly confident DVRs. Our approach contrasted with other previously delineated methods for detecting APOBEC3 RNA editing sites as it captures not only RNA editing events but also considers the consistency and magnitude of these events [19, 20]. A schematic representation of this analytical procedure is depicted in Fig. 1C and the outputs at different stages are shown in Fig. S1B. We compared the results for A3B-induced RNA variant (RV) and DVR identification using our analytical pipeline outlined in Fig. 1C to the original rMATS-DVR method [20] and found a reduced false-positive overlap with pre-existing DNA SNVs present in T-47D genomic DNA (GSE193226) (Supplementary Tables 1, 2) [22].

To ascertain if our analytical workflow could pinpoint DVRs ascribed to augmented A3B activity, we processed T-47D cell samples with and without doxycycline exposure (Fig. 1D). We verified the induction of A3B at both the mRNA and protein levels following doxycycline treatment and extracted DNA and RNA samples for next generation sequencing and DVR assessment. The first round of analysis with our DVR detection pipeline revealed a marked rise in DVRs in poly-A enriched RNA following A3B induction, this included a build-up of A > G(I) DVRs and an even more pronounced accumulation of C > U DVRs (Fig. 1E, F). In addition, closer inspection of these DVRs finds the editing levels for most of the C > U DVRs were increased due to prolonged exposure to ectopically-expressed A3B, which was a phenomenon not found for A > G(I) DVRs (Fig. S1C). These results suggest that the C > U editing sites detected were related to additional A3B catalytic activity by doxycycline induction.

The RNA preparation technique employed will dictate the RNA categories that will be sequenced. Initially, we employed poly-A enrichment, predominantly capturing mRNA. Next, to gain a more comprehensive perspective on RNA editing, we compared next generation sequencing of poly-A enriched RNA with a ribosomal RNA depletion protocol that facilitated the incorporation of non-polyadenylated RNA variants into our DVR analysis. Post 72 h of A3B induction, we demonstrated that the ribosomal RNA depletion technique augmented DVR detection by ≈3-fold (Fig. 1E, F). The predominant DVRs discerned in the ribosomal-depleted RNA stemmed from A > G(I) editing that was increased by ≈6-fold compared to the poly-A enriched RNA. The ribosomal RNA depletion-enriched samples also manifested heightened C > U DVRs, by ≈0.5-fold relative to poly-A enriched RNA.

Employing the 72-h induction and ribosome depletion approach, which optimally sensitised DVR detection, we examined DVRs produced by the A3B** construct as a negative control. We ascertained that the induction levels of both the mutant and native A3B were comparable (Fig. 1B). However, the expression of the enzymatically inert A3B** significantly diminished DVR detection compared to its active counterpart (Fig. 1E, F). Notably, C > U variant production was profoundly affected relative to A > G(I) variants. The induction of the inactive A3B** yielded a mere two C > U DVRs, in contrast to the several hundred C > U DVRs observed with the wild-type A3B (Fig. 1E). To investigate the potential non-specific effects of the induction agent doxycycline, we conducted control experiments using T-47D cells treated with 1 μg/ml doxycycline compared to a DMSO vehicle control. The results confirmed that the observed increase in detected DVRs is primarily attributable to the induction of exogenous protein, as cells treated with doxycycline alone showed only a minimal number of DVRs (Fig. S2). By conducting differential expression analyses on the RNA-seq results, we also investigated whether the ectopic induction of A3B or A3B** affects the intrinsic APOBEC or ADAR families of RNA editors. As shown in Fig. S3A, for all the genes investigated none were significantly altered except for APOBEC3F which was up-regulated but to negligible levels compared to intrinsic and ectopic A3B. This result affirms that the C > U DVRs were unlikely to be attributed to activities from other members of APOBEC family, and that the A > G(I) DVRs were unlikely to be linked to potential transcriptional regulation of ADAR family due to A3B induction (Fig. S3B, C). Collectively, our findings underscore that the C > U editing sites discerned resulted from prolonged A3B induction, and the C > U DVRs identified through our refined detection method hinge on the catalytic efficacy of the doxycycline-induced A3B.

### Validation and characterisation of DVRs

**Fig. 2:**
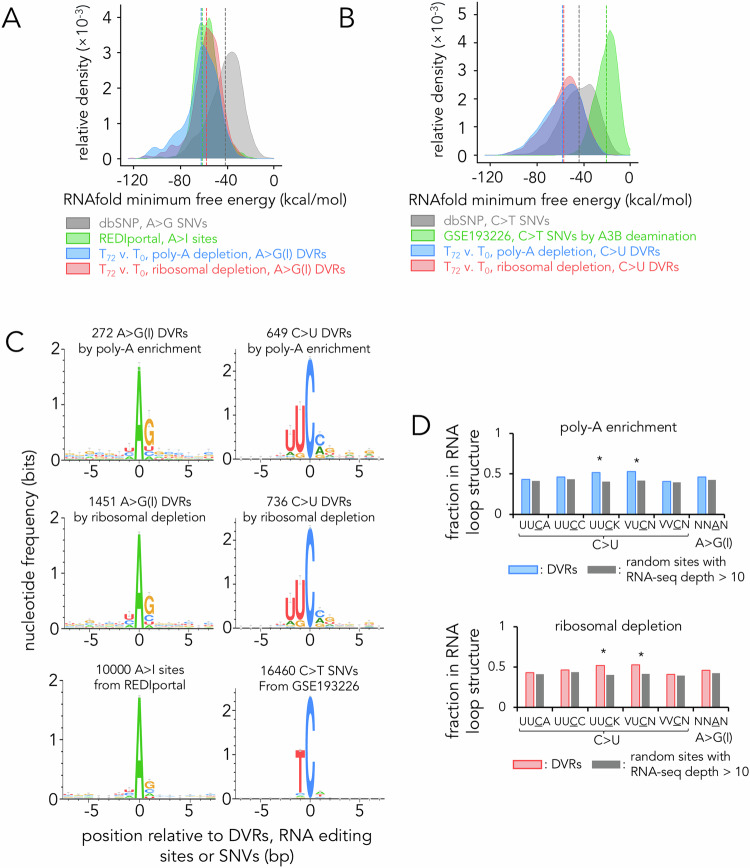
Characterisation of A3B-mediated DVRs. **A** RNA folding minimum free energy predicted by RNAfold for A > G genomic DNA SNVs, known RNA A > I sites and A > G(I) DVRs identified in this study. **B** RNA folding minimum free energy predicted for C > T genomic DNA SNVs, known A3B-mediated genomic DNA C > U editing sites and C > U DVRs identified in this study. Random sampling was applied for SNVs from dbSNP database (*n* = 100,000) and A > I sites (*n* = 10,000) from REDIportal. **C** Fraction of indicated types of DVRs or control sites within loop structure predicted by RNAfold. For random control, *n* = 10,000 random sites with RNA-seq read depth of greater than 10 in T-47D transcriptome were sampled. Error bars denote SD. * denote *p* < 0.05 using Z-test. **D** Consensus sequence logo plots for sequences centred at editing sites were generated by WebLogo3.

To ensure that RNA C > U DVRs we identified were not inaccurately attributed to genomic DNA SNVs we used the RNAfold algorithm [23] to estimate the free energy linked to RNA folding of 100 nucleotides of sequence surrounding the DVRs. We then predicted the relationship between RNA structure and location of the base modification, specifically if the edited sites were located within double-stranded base-paired stems or single-stranded loop structures. Our analysis revealed that sequences adjacent to A > G(I) DVRs exhibited a comparable RNA folding energy distribution as curated RNA A > I edited sites [24], but differed from the genomic A > G SNVs listed in dbSNP [25], which exhibited less energetically favourable folding (Fig. 2A). Sequences adjacent to C > U DVRs also followed the A > G(I) pattern, with sequences adjacent to the RNA C > U edited sites exhibiting more energetically favourable folding than the known genomic C > T SNVs (dbSNP) or previously documented genomic A3B C > T edited sites (GSE193226, Fig. 2B) [22].

We charted the distribution of nucleotides adjacent to the DVRs (Fig. 2C). For A > G(I) DVRs, the patterns of surrounding nucleotides closely mirrored known RNA A > I sites [7, 26, 27], validating the DVRs’ origin from RNA editing, presumably by the ADAR family of RNA editors. Conversely, for C > U DVRs, there was a clear predilection for the UUCV (V = A, C or G) sequence motif, a contrast to the favoured TCW (W = A or T) sequence motif typical detected for genomic DNA sites edited by A3B (GSE193226; Fig. 2C) [22]. Detailed examination of the RNAfold-predicted structures revealed a marked enrichment of the C > U DVRs in predicted single-stranded RNA hairpin structures (Fig. 2D). These single-stranded RNA hairpins are more susceptible to base deamination, which is in concordance with previous studies with A3B [28, 29] and A3B’s closely related homologue A3A [7, 30]. Heightened editing within predicted hairpin structures was noted for UUCK > UUUK (K = G or T) and VUCN > VUUN (V = A, C, or G, N = A, U, C or G) DVRs, suggesting a specific affinity for the nucleotides surrounding the C > U DVRs. To further investigate the structural context of C > U DVRs, we utilised the RNAFold tool to analyse the RNA secondary structures. This analysis demonstrated that the preferred sites for A3B editing predominantly feature predicted hairpin structures, potentially enhancing enzyme accessibility (Fig. S5). These results indicate that A3B’s RNA editing activity is influenced by both the sequence and structural context.

**Fig. 3:**
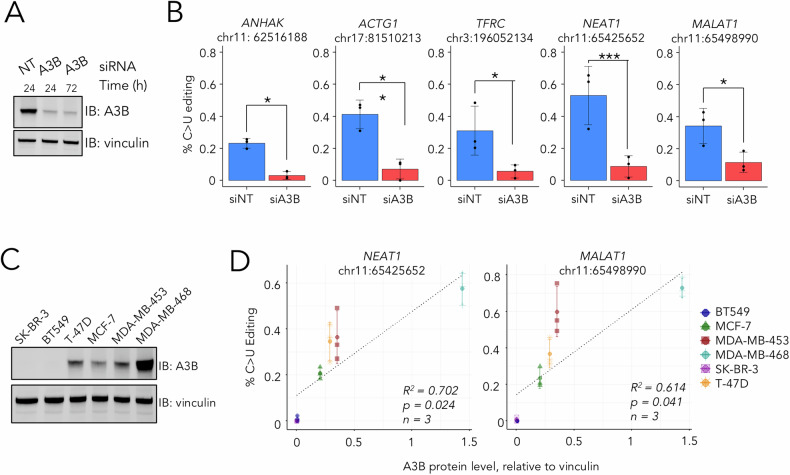
Validation of A3B dependency for selected C > U DVRs. **A** Immunoblotting confirming effective knockdown of A3B in T-47D cells using A3B-targeting siRNA. **B** Plots showing results from the ddPCR determination of C > U editing at indicated sites. Columns showing average of *n* = 3 independent experiments, and error bars for SD. *, ** and *** denotes *p* < 0.05, 0.01 and 0.001 using Student’s T test. **C** Immunoblots displaying the expression levels of A3B across six breast cancer cell lines. Band intensities for A3B and the loading control (vinculin) were quantified using LI-COR Odyssey DLx. **D** Scatter plot correlating the levels of C > U editing at NEAT1 or MALAT1 with A3B expression levels across the six cell lines. C > U editing was quantified using droplet ddPCR. Data denotes average of *n* = 3 independent experiments, and error bars for SD. Linear regression analysis was conducted, and correlation coefficients are provided.

To further validate the physiological occurrence of the C > U DVRs initially discovered through inducible expression, we selected five DVRs demonstrating significant doxycycline-induced editing (Fig. S4). We used droplet digital PCR (ddPCR) to measure the effect of A3B depletion on the editing levels of these selected sites following treatment of the parent T-47D cells with A3B siRNA (Fig. 3A, B). A statistically significant and substantial reduction in the editing frequencies at these sites was detected following the knockdown of A3B, consistent with the observed C > U DVRs being directly attributed to the endogenous A3B. We also expanded our analysis to include breast cancer cell lines with varied A3B expression and minimal A3A interference [11], and quantified C > U editing of DVRs within NEAT1 and MALAT1, the two transcripts with highest degree of C > U editing, using ddPCR. Our results reveal a strong correlation (R² > 0.6, *p* < 0.05; Fig. 3C, D) between A3B protein levels and editing frequencies, confirming A3B’s role in RNA editing across different cell types. In sum, these findings collectively underscore the endogenous role of A3B in mediating C > U RNA editing.

**Fig. 4:**
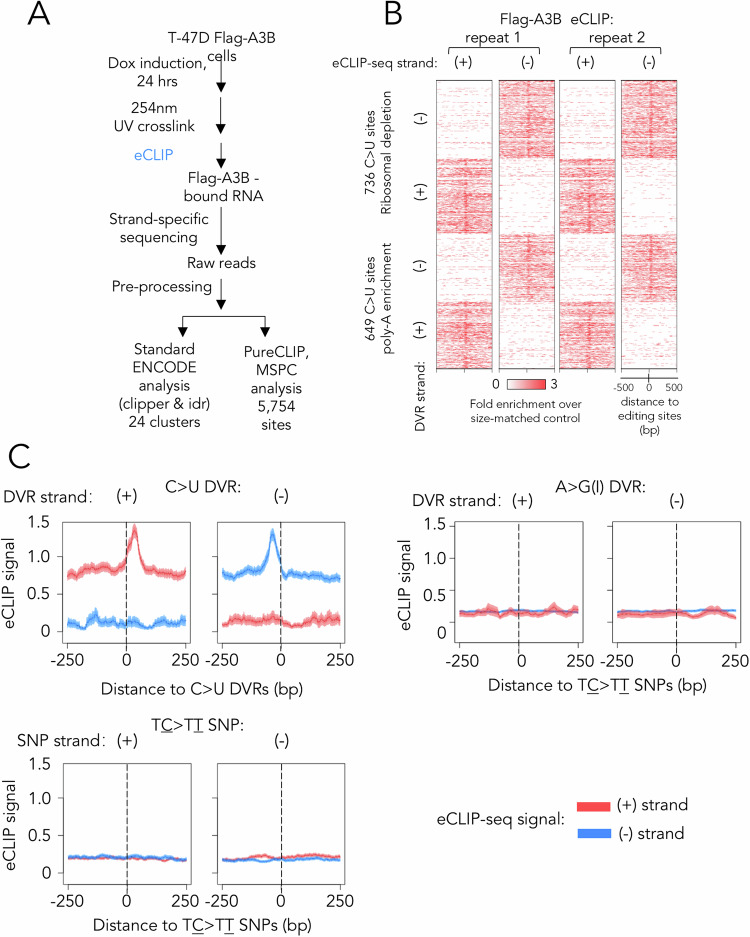
Validation of A3B-mediated C > U DVRs using eCLIP-seq. **A** Schematic of the eCLIP-seq and analysis pipeline in this study. **B** Heat maps showing eCLIP-seq signals in regions flanking all C > U DVRs identified upon A3B induction. Results from *n* = 2 eCLIP-seq signals from different strands are shown separately. **C** Profile plot showing eCLIP-seq signals in regions flanking all C > U DVRs identified upon A3B induction. For comparison, eCLIP-seq signals flanking A3B-mediated TC > TT SNPs identified from GSE193226 are shown. For (**B**, **C**), data represent fold enrichment of eCLIP-seq signal over size-matched control.

To further validate the role of A3B in the generation DVRs, we employed eCLIP-seq to map A3B-bound RNA sites in T-47D cells with high precision. We then scrutinised the data to determine whether A3B binding was enriched at DVR sites. By using this method we aimed to ascertain whether the C > U DVRs result from RNA binding of A3B, where A3B binding sites were identified by next generation sequencing following isolation of RNA that was UV cross-linked to the induced Flag-tagged A3B (Fig. 4A). We processed the sequencing data using both the standard ENCODE protocol [31] and the PureCLIP programme [32]. The enriched eCLIP-seq signals were positioned immediately after C > U DVRs (Fig. 4B, C). This enrichment stems from the hindrance of reverse transcription by the crosslinked A3B:RNA complex during library preparation, affirming that A3B binds the RNA sites where C > U editing occurred. In contrast, there was no A3B eCLIP-seq signal enrichment around or adjacent to the A > G(I) DVRs, suggesting RNA binding or editing by A3B was not directly involved in generating these DVRs (Fig. 4C). Also, no enrichment of eCLIP-seq signal was found near previously identified A3B-mediated TC > TT genomic SNPs (Fig. 4C). While eCLIP-seq identified enrichment of A3B binding signal at C > U DVRs, not all C > U DVRs were overlapping with A3B binding sites identified by eCLIP-seq. We interpreted this as a reflection of the dynamic nature of A3B action, where A3B briefly binds, edits the RNA, then disengages and in the absence of repair mechanisms leaves a long-lived DVR. Further analysis of sequences around the A3B-associated eCLIP-seq clusters revealed a heightened frequency of the UUCV and UUCK motifs, previously pinpointed through RNA-seq and RNA-fold analysis, at A3B binding sites (Fig. S6). Overall, the evidence from our eCLIP-seq analysis were consistent with the output from our RNA-seq analysis pipeline comparing catalytically active and inactive A3B. Together with the RNAfold analysis these data indicated that the C > U DVRs we detected were highly likely to result from the RNA editing activity of A3B, and not from editing of genomic DNA.

### A3B edits RNA in a selective manner

**Fig. 5:**
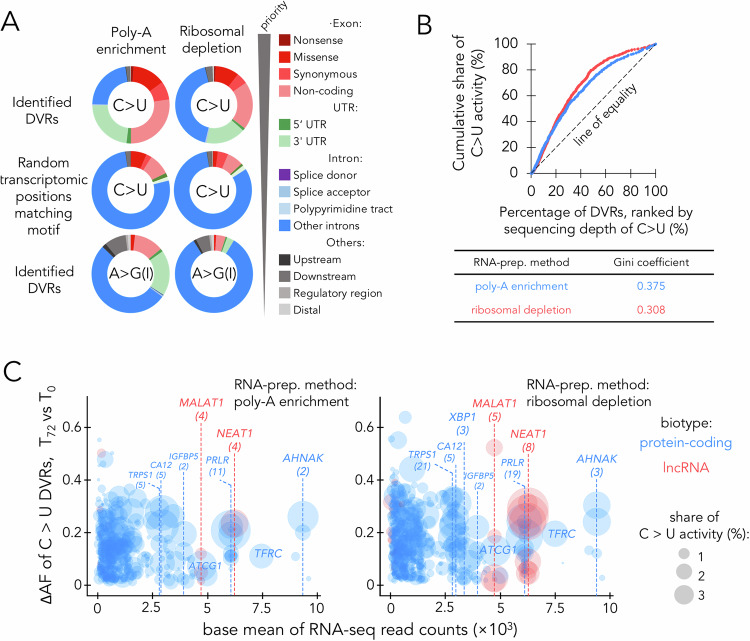
A3B edits RNA in a selective manner. **A** Distributions for editing sites across different types of RNA features by type of RNA editing. For variant with multiple editing consequences, only the one with highest impact predicted by VEP was counted. The rank of impact was shown on the right. **B** Lorenz curve showing the level of inequality distribution of A3B’s activity across identified C > U DVRs, and the calculated Gini coefficients for different sample group. **C** Bubble plot showing distribution of A3B activity across identified C > U DVRs. Vertical axis shows difference in faction of U bases, and horizontal axis shows level of gene expression for edited genes (as normalised read counts by DESeq2, also referred to as base mean), with size of the bubble denote share of total A3B’s C > U editing activity. The ‘share of C > U activity (%)’ is calculated as the ratio between C > U mutations at each DVR and the total C > U mutations across all DVRs, both of which were adjusted using per million mapped reads. Numbers within parenthesis denote the number of DVRs within indicated genes.

We subsequently examined the impact of the identified A3B-mediated DVRs on transcripts. Initially, we utilised the Variant Effect Predictor [33] to annotate the RNA locations and the potential consequences of the DVRs within the T-47D transcriptome. Figure 5A depicts a significant enrichment of C > U DVRs in mRNA exons and 3’UTRs, when compared to un-induced T-47D control transcriptome positions matching the A3B C > U DVR sequence motif. Among the exonic DVRs, events in untranslated exons were predominant, with missense mutations in the resulting protein sequence being the next most common. Conversely, A > G(I) DVRs were primarily intronic, aligning with prior findings [7, 26, 27]. We also found A > G(I) DVRs were associated with transcripts showing significantly (FDR < 0.05) altered levels following A3B induction, contrasting with C > U DVRs that were generally associated with RNAs showing no or low levels of altered expression (Fig. S7). This indicated that unlike A > G(I) editing which can alter mRNA splicing or stability, C > U RNA editing does not significantly impact RNA processing or degradation.

To discern any potential selectivity in A3B’s activity, we quantified the distribution inequality of its C > U activity using the Gini coefficient [34]. We constructed Lorenz curves, plotting the cumulative proportion of C > U, indicative of A3B activity, against the rank of DVRs based on the depth of C > U mutations from RNA-seq experiments (Fig. 5B). In these plots, a straight diagonal line signifies an even distribution of A3B generated C > U DVRs across the transcriptome. Any deviation from this line suggests an unequal distribution of A3B activity, with the curve’s increasing curvature highlighting a greater degree of inequality. The Gini coefficient, derived from the plot, ranges from 0 (even distribution) to 1 (maximum inequality). For A3B C > U editing, the Gini coefficient was determined to be 0.375 and 0.300 for RNA samples processed using ribosomal depletion and poly-A enrichment methods, respectively. This points to a substrate bias in A3B activity, indicating a preference for editing specific RNAs. On closer analysis, approximately 40% of the A3B edited C > U DVRs were concentrated in the top 23 edited genes for the ribosomal-depleted samples and the top 44 genes for poly-A enriched samples (Fig. 5C). Specifically, for RNA processed via the ribosome depletion method, the editing of two non-coding RNAs, NEAT1 and MALAT1, constituted a significant fraction of the overall activity of A3B, accounting for 7.3% and 1.8%, respectively (Fig. 5C). For the C > U DVRs identified in A3B-GFP samples, the majority exhibited negligible C > U editing activity in cells expressing the inactive A3B**-GFP (Fig. S8). These findings underscore A3B’s substrate selectivity within the T-47D cell transcriptome and highlight specific hotspots for A3B-mediated C > U editing.

### A3B binds and edits NEAT1 and MALAT1

**Fig. 6:**
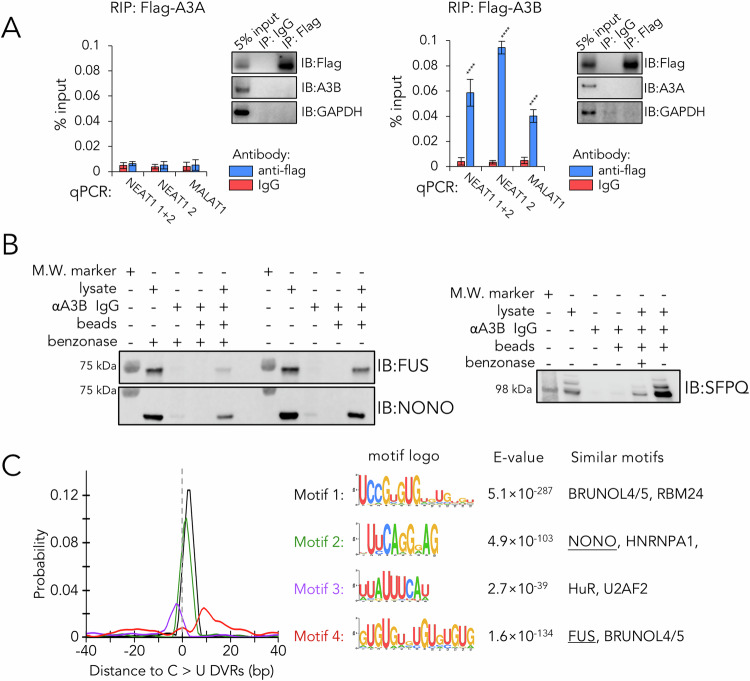
NEAT1 and MALAT1 bind to A3B. **A** Results from RIP-qPCR with co-immunoprecipitation of over-expressed flag-tagged A3A (left) or A3B (right) in T-47D cells. Data denotes mean of *n* = 3 biological replicates, and error bars for SD. **B** Representative results for co-immunoprecipitation of A3B in T-47D cells. **C** Analysis of motif enrichment at ±40 bp sequences centred at all C > U DVRs identified in this study. Profiles showing probability of top-ranking sequence motifs were shown on the left, and the details for the top-ranking sequence motifs were shown on the right.

The pronounced enrichment of C > U editing by A3B on NEAT1 and MALAT1 RNAs prompted us to postulate a model of selective binding of A3B to these lncRNAs. The eCLIP-seq results, confirmed a marked enrichment of A3B-crosslinked RNA fragments to both *NEAT1* and *MALAT1* RNAs. These data revealed read clusters with heightened signals compared to the control size-matched input RNA, in this case suggesting stable A3B binding (Fig. S9). Notably, some clusters displayed C > U DVRs, reinforcing the notion that A3B binding at these loci could result in RNA editing. Subsequent RNA immunoprecipitation (RIP) assays in 293 T cells overexpressing flag-tagged A3A and A3B demonstrated co-precipitation of NEAT1 and MALAT1 RNA variants with flag-tagged A3B, but not A3A (Fig. 6A). We have previously employed proximity biotinylation-based labelling (BioID) to pinpoint A3B’s binding partners across several breast cancer cell lines [22]. Mass spectrometry analysis from that study identified several A3B interactors that were also NEAT1 and MALAT1 RNA binding proteins, notably nuclear and/or paraspeckle proteins FUS, NONO, and SFPQ [22, 35]. We corroborated these findings by demonstrating co-immunoprecipitation of A3B with FUS, NONO, and SFPQ in T-47D cells. This association persisted after removal of nucleic acids by benzonase treatment, suggesting direct protein-protein interactions between A3B and these proteins (Fig. 6B). For further validation, we analysed sequences flanking all identified C > U DVRs in T-47D cell’s transcriptome using the MEME package [36], which revealed high enrichment for NONO and FUS binding motifs in proximity to C > U DVRs, (Fig. 6C). Collectively, our results propose a model where A3B interacts and edits NEAT1 and MALAT1 lncRNAs that is mediated through their associated lncRNA protein partners.

Given the documented regulation of A3B by the oestrogen receptor (ER) [16], we explored the influence of ER on A3B-mediated RNA editing by transiently knocking down *ESR1* in T-47D cells and assessed the activity of A3B on NEAT1 and MALAT1 using ddPCR. Knockdown of ESR1 effectively reduced ERα expression but did not affect the expression of NEAT1, MALAT1 or A3B (Fig. S10A, B) and no significant changes in C > U editing levels were detected (Fig. S10C). To further investigate the regulatory effect of oestrogen on A3B’s interactions with NEAT1 and MALAT1, we conducted RIP experiments following estradiol treatment in T-47D cells. These data show that activation of the ER by estradiol does not significantly impact A3B’s binding to these lncRNAs following estradiol treatment of T-47D cells (Fig. S10D, E). These findings collectively suggest that A3B’s RNA editing activity and its interactions with NEAT1 and MALAT1 are regulated through mechanisms distinct from direct ER signalling.

### NEAT1 and MALAT1 regulate A3B’s catalytic activity

**Fig. 7:**
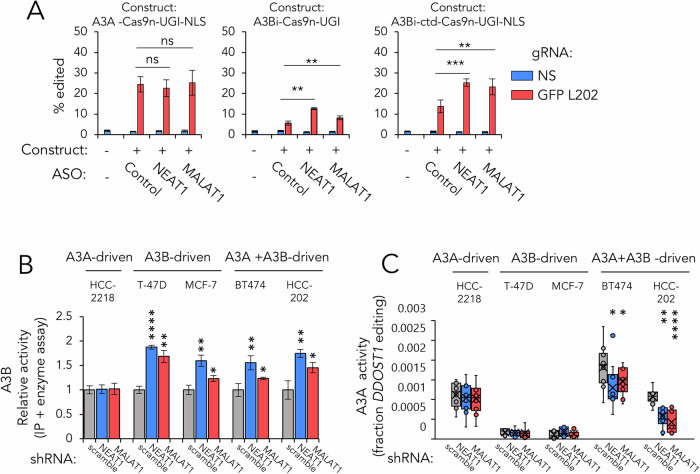
NEAT1 and MALAT1 regulate A3B activity in cells. **A** Quantification of the effect of lncRNA depletion using ASOs on APOBEC3 enzymes’ activity in cells with the APOBEC-Cas9n reporter system. 293 T cells were used in this study. Data denotes mean of *n* = 3 biological repeats, and error bars for SD. **B** Quantification of A3B activity in cells using the BspH1 biosensor with immunoprecipitated A3B complex in breast cancer cell lines stably expressing shRNAs. Bar graph was shown with data normalised using scramble shRNA control. Data represent mean of *n* = 3 biological repeats, and error bars for SD. **C** Quantification of A3A activity in cells by level of *DDOST1* 558 C > U editing, measured by ddPCR. Box plot representing *n* = 9 biological repeats. *, **, ***, **** and ns denote *p* < 0.05, 0.01, 0.001, 0.0001 and non-significant using two-tailed Student’s *T* test, evaluating difference against scramble control shRNA-expressing samples.

NEAT1 and MALAT1 non-coding lncRNAs and their protein-binding partners are frequently overexpressed to similar level in different cancer types [37, 38]. We postulated that interactions between A3B and NEAT1/MALAT1 ribonucleoproteins (RNPs) might modulate A3B’s catalytic activity, either by acting as suicide substrates or by sequestering A3B. To explore this, we utilised a previously established fluorescent reporter system to gauge the enzymatic activity of APOBEC3 enzymes in 293 T cells [39]. We then assessed the implications of acute NEAT1 and MALAT1 depletion using antisense oligonucleotides (ASOs), an approach frequently used to deplete non-coding RNAS (Fig, S11A, B). We introduced plasmid constructs encoding Cas9n fusion proteins with A3A, A3B, and A3B’s CTD into 293 T cells, alongside an eGFP variant containing a T > C mutation at L202 (L202S, TTA > TCA) that ablates fluorescence and creates a potential A3B editing site. A3B editing of the L202 site generates a functional GFP, which can be quantified using fluorescence microscopy [39]. Notably, while acute depletion of NEAT1 and MALAT1 remained inconsequential for the editing activity of the A3A fusion protein, the activities of both full-length A3B and CTD-truncated A3B were significantly enhanced post depletion of NEAT1 or MALAT1 expression (Fig. 7A). This evidence supports our model that NEAT1 and MALAT1 lncRNAs act as negative modulators of A3B activity.

To determine the impact of prolonged NEAT1 and MALAT1 depletion on APOBEC3 enzymes’ activity, we stably expressed shRNA targeting these lncRNAs and confirmed the efficacy of the shRNAs through RT-qPCR (Fig, S11C, D). We included five breast cancer cell lines, each with well-characterised A3A and A3B expression profiles [16], to investigate the activity of intrinsic A3B. These cell lines were specifically chosen to represent diverse biological contexts: HCC2218, which only expresses A3A; T-47D and MCF-7, which exclusively express A3B; and BT474 and HCC-202, where both A3A and A3B are highly expressed. This selection enables a comprehensive analysis of DNA deamination driven by A3A, A3B, or a combination of both enzymes. A3B activity was measured by immunoprecipitating the A3B complex and measuring enzymatic function using a DNA deaminase biosensor incorporating BspHI restriction enzyme [40]. In the four A3B expressing cell lines, NEAT1 and MALAT1 depletion led to an enhanced enzymatic activity of A3B, underscoring that sustained suppression of their expression augments A3B activity (Fig. 7B). However, this was not observed in HCC2218 cells, a line with minimal A3B expression.

Recent findings have highlighted a negative feedback mechanism governing A3A and A3B activity [41], We explored whether the sustained increase in A3B activity, following loss of NEAT1 and MALAT1, might affect A3A function. We assessed level of C > U editing at the *DDOST1* C558 site (C558U, UCG > UUG) utilising ddPCR, a recognised technique designed for measuring A3A activity but not for other APOBEC3 family members since it was identified to be A3A-specific [30]. Our results revealed that prolonged depletion of NEAT1 and MALAT1 did not alter A3A activity in the A3A-exclusive HCC2218 cells, but did diminish A3A activity in cells with high expression of both A3A and A3B (BT474 and HCC202) (Fig. 7C). The concomitant increase in A3B activity following NEAT1 and MALAT1 removal in these cells (Fig. 7B) suggests a model where release of active A3B in some cells restrains of A3A activity.

To investigate the clinical implications of NEAT1 and MALAT1 regulation on A3B activity, we analysed The Cancer Genome Atlas (TCGA) datasets for breast cancer (BRCA) and lung adenocarcinoma (LUAD) [42], cancers that are known for having significant APOBEC-mediated mutational signatures and A3A/A3B aberrant overexpression [12]. Initial analysis revealed no direct correlation between NEAT1/MALAT1 and A3A/A3B transcript levels (Fig. S12A, B). We then stratified patients based on high (top tercile) or low (bottom two terciles) gene expression levels, giving four distinct groups to assess genomic cytidine deamination patterns potentially driven by these enzymes (Figs. S12C, D, S13). We evaluated mutational burden using non-negative matrix factorisation (NMF) to quantify APOBEC-related mutations, specifically single base substitutions (SBS) 2 and 13 [43]. Interestingly, in tumours predominantly expressing A3B, high NEAT1 or MALAT1 expression significantly correlated with reduced levels of the APOBEC-mediated mutation signatures, aligning with our hypothesis that NEAT1 and MALAT1 can regulate A3B activity by sequestration (Fig. S13). Conversely, in patients co-expressing A3A and A3B, higher levels of NEAT1 or MALAT1 were associated with increased APOBEC-associated mutation signatures, suggesting that in these contexts, the sequestration of A3B may lead to enhanced A3A activity due to its unimpeded deaminase function (Fig. S13). These findings underscore the complex regulatory dynamics between NEAT1/MALAT1 and APOBEC enzymes and illustrate a link between NEAT1/MALAT1 expression and A3A/A3B mutational burden in cancer patients.

## Discussion

Two members of the APOBEC3 family (A3A and A3G) are the only members that have so far been reported to have bona fide RNA editing activity. The A3B member of this family is reported as key molecular driver of cancer mutations, however, its high sequence and structural similarity to A3A has also suggested this family member may have RNA editing activity [15, 41, 44]. Characterisation of the RNA editing functions of A3B in human cancers has remained a challenge primarily due to interference from the genomic DNA cytidine deamination activity of A3B [6] and, depending on the cellular context, background RNA editing from other APOBEC3 family members - most notably A3A [7, 45]. Consequently, a better understanding of A3B-mediated RNA editing necessitated the development of an effective cellular models, the implementation of more stringent controls, and the enhancement of analytical methodologies.

In the present study, we report the identification and mapping of A3B-mediated RNA editing sites by integrating a lentiviral inducible expression breast cancer model with next-generation sequencing and an analysis pipeline to detect and map RNA edits (DVRs). By combining WGS from controls with inducible expression of A3B, or a catalytically dead A3B, we developed a sensitive method that could: (1) detect low-level editing events and (2) isolate statistically significant DVRs that were solely attributed to elevated A3B expression, while managing interference from other APOBEC3 family members. Using this model, we were able to locate A3B RNA editing sites in the T-47D breast cancer transcriptome, strengthening the predictions that A3B can operate as an RNA-editing enzyme in cancer cells.

Unlike A3B’s genomic editing sites which are typically repaired by base excision repair (BER) [46], the uracil bases resulting from A3B’s RNA editing are retained. In a previous study with a BER-deficient cellular model we unveiled A3B’s preference for genomic regulatory regions such as enhancers and promoters [22]. In contrast, here we discovered that the RNA edited sites of A3B had a higher incidence of coding region mutations, suggesting A3B activity could be a significant source of amino acid alterations contributing to tumour adaptation and drug resistance [47, 48]. We also found editing sites in mRNA 5’- and 3’-UTRs, which could influence translation initiation, stability, and localisation of RNAs. These observations and the additional detection of A > G(I) DVRs in T-47D cells, in conjunction with A3B induction, raises questions regarding the functional impact of RNA editing by A3B. While this study presents insights into the generalised editing activity of A3B across transcriptomes, the absence of RNA-seq analysis on A3B-silenced cells limits our ability to explore its differential editing effects. Future investigations comparing parental and silenced cell conditions would be instrumental in clarifying the direct roles of endogenous A3B in RNA editing. Elevated A3B expression did not significantly influence ADAR deaminase expression, nor did it result in increased binding of A3B at A > G(I) sites or show proximity between C > U and A > G(I) coordinates. Therefore, the regulation of A > G(I) editing by A3B is most likely to be indirect. While the increase in A > I editing following the induction of the enzymatically inactive A3B**-GFP variant comparing to the effect of doxycycline alone suggests potential contributions from non-enzymatic functions of A3B or other elements of the lentiviral system, and delineating the precise origins of these A > G(I) edits will require further targeted investigations.

Recent studies have consistently highlighted A3B specific substrate preferences when acting on DNA. A3B shows a strong preference for cytosines within single-stranded DNA structures [49]. This specificity allows the enzyme to target sequences protruding from the DNA double helix, a process facilitated by A3B’s structural ability to adapt to such bends or loops [50]. Notable targets include the lagging DNA synthesis strand [51–53], loop regions of DNA [22], and ssDNA intermediates during recombination and repair [54–56]. Our study extends the understanding of A3B’s substrate recognition, suggesting preference for secondary structural motifs in RNA such as hairpins and bulges, which aligns with a recent study shown A3B’s preference for cytosines in stable, short, four-nucleotide DNA hairpin loops [28]. Since in vitro studies show that A3B binds RNA with less affinity than DNA, its activity toward specific RNA structures, especially secondary motifs such as hairpins and bulges that expose protruding nucleotides, merits further investigation.

We also showed that both poly-A enrichment and ribosome RNA depletion methods of library production successfully identified DVRs, but these techniques revealed preferences in the types of A3B-mediated DVRs detected. This comparison particularly illuminated a regulatory mechanism of A3B by long non-coding lncRNA, specifically by NEAT1 and MALAT1, which are abundant components of paraspeckles and nuclear speckles [37, 38, 57]. Our results align with structural and sequence homology data that predict the A3B NTD should bind to RNA [45]. Our findings suggest the involvement of NEAT1 and/or MALAT1 and A3B in an RNP complexes. However, although through homology the NTD of A3B is predicted to bind RNA, our experiments using the CRISPR-Cas9n reporter system demonstrate that the CTD alone can also manifest RNA editing function, and this can be influenced by cellular NEAT1/MALAT1 levels. This is consistent with a previous report that the deamination activity of A3B is attenuated in an RNA-dependent manner and activated in cell lysates following RNAse A treatment [45].

Some human tumour cells tolerate high levels of A3B, however, sustained high expression of A3B is toxic to many cancer cells and normal tissue in vitro and in vivo [11, 58]. Experiments in syngeneic cancer models found that A3B-catalysed mutations induce adaptive immune responses that inhibited tumour growth [59]. Overall, evidence is emerging for A3B and the other APOBECS having diverse roles that can be both disadvantageous and beneficial to tumours [47, 60–62]. Genome sequencing of prolonged cell culture models reveals intermittent APOBEC-driven mutation bursts, interspersed with extended inactive phases. These intermittent bursts contribute to significant mutation burden and promote sub-clonal heterogeneity in tumours [63]. The frequency of these bursts in vivo remains undetermined and challenging to verify. Moreover, the mechanisms underlying these fluctuations are unclear, as they do not correlate with APOBEC RNA levels [63], indicating possible post-transcriptional regulation of APOBEC protein expression or activity. Here we speculate that the cancer-associated lncRNAs NEAT1 and MALAT1 may modulate A3B activity, possibly by sequestration or serving as suicide substrates. In addition, these lncRNAs are involved paraspeckle and nuclear speckle interactions [37, 38, 57] which may also influence the cellular localisation and activity of A3B. These observations highlight a promising research direction for understanding A3B regulation and its implications in tumorigenesis.

Analyses of patient tumours and pre-clinical models suggest that APOBEC expression or mutation burden could predict treatment outcomes for therapies including platinum-based, immunotherapy, or targeted approaches [64]. Although APOBEC status may serve as a prognostic biomarker, defining “APOBEC-positivity” clinically is still pending. Elevated NEAT1 and MALAT1 levels correlate with A3B activity modulation, indicating their potential as biomarkers for monitoring A3B enzymatic activity in cancer. Supported by TCGA data, NEAT1 and MALAT1 expressions could predict A3B activity, aiding in evaluating cancer progression and response to A3B-targeted therapies. Furthermore, while A3A predominantly drives genomic mutations in cancer, A3B has a lesser mutagenic capacity. Loss of NEAT1/MALAT1 reduces A3A activity while activating A3B, complementing findings that A3B negatively regulates A3A-driven mutagenesis [41]. Preliminary clinical data show that higher NEAT1 and MALAT1 expressions correlate with reduced A3B and increased A3A activities in cancers like breast and lung, suggesting their role in enzymatic balance between A3B and A3A, affecting mutation dynamics and cancer progression. Thus, NEAT1 and MALAT1 could predict A3A activity in cells and patients [64, 65]. However, the exploratory nature of these findings and data limitations mean further research is needed to confirm their roles as biomarkers and therapeutic implications. It is important to note that our analyses of the TCGA dataset cannot definitively exclude potential influences from other APOBEC enzyme members, which may also contribute to observed mutational signatures. Comprehensive studies in larger patient cohorts with detailed genomic analysis are essential to confirm the regulatory roles of NEAT1 and MALAT1, contributing to a comprehensive understanding of how APOBEC3 enzymes promote cancer evolution by causing genome and transcriptome heterogeneity.

## Materials and Methods

Method details are provided in the supplementary text.

## Supplementary information

Supplemental Material

Supplemental Figure 1

Supplemental Figure 2

Supplemental Figure 3

Supplemental Figure 4

Supplemental Figure 5

Supplemental Figure 6

Supplemental Figure 7

Supplemental Figure 8

Supplemental Figure 9

Supplemental Figure 10

Supplemental Figure 11

Supplemental Figure 12

Supplemental Figure 13

Supplemental List 1

Supplemental List 2

## Data Availability

The high through-put sequencing data, together with processed data files, was available on GEO database with accession number GSE245700 and GSE245701.
