# Supplementary material for: Characterisation of APOBEC3B-Mediated RNA editing in breast cancer cells reveals regulatory roles of NEAT1 and MALAT1 lncRNAs
Source: Oncogene. 2024 Sep 25;43(46):3366–77. doi: 10.1038/s41388-024-03171-5 (PMC11554567; doi:10.1038/s41388-024-03171-5)
Supplement: Supplementary file 1 — Supplemental Material [file 41388_2024_3171_MOESM1_ESM.docx]

# Supplemental Methods

## Cell culturing and cell line preparation

The T-47D, HCC2218, MCF-7, BT474, HCC202, SK-BR-3 and MDA-MB-453 and MDA-MB-468 human breast cancer cell lines were obtained from ATCC. Lenti-X 293T cells were obtained from Clonetech. T-47D cells were maintained in RPMI-1640 medium supplemented with bovine insulin, and Lenti-X 293T cells were cultured with DMEM medium. Both media were supplemented with 10% v/v FBS (PAA) and 0.5% v/v pen/strep. All cells were cultured at 37 ºC with 5% CO2, were validated using STR and were regularly tested for mycoplasma contamination. To generate stable inducible cell lines, exponentially growing T-47D cells were transfected with lentivirus in the presence of 10 µg/ml polybrene. After a transduction period of 48 hours, selection was carried out using medium supplemented with 4 µg/mL puromycin (Gibco). Transduction efficiency was monitored by GFP expression as well as quantitative RT-PCR following induction of sample cells with doxycycline. After verification, lentivirus cassettes were maintained by culturing cells with 4 µg/mL puromycin. For the induction of lentiviral protein expression, cells were exposed to full RPMI-1640 culture medium supplemented with 100 ng/mL doxycycline (Sigma).

## Whole Genome Sequencing

Exponentially growing T-47D cells were cultured in T175 flasks and were harvested using trypsin digestion. Genomic DNA was extracted using a Qiagen Genomic-tips 500/G kit. Sequencing libraries for whole genome sequencing with DNA nanoball (DNB) technology were constructed (Beijing Genomics Institute (BGI) Inc., Hong Kong). Sequencing was carried out using BGISEQ-500 sequencer with a mean sequencing coverage of greater than 30× for each of the samples using 2 × 150 bp configuration. Sequence reads from WGS was aligned to GRCh38 genome assembly using Burrow-Wheelers aligner [1].

## RNA sequencing and transcript quantification

Total RNA were extracted from T-47D cells using the MagNA pure 96 platform (Roche), and RNA integrity number (RIN) was determined by BioAnalyzer 2100 (Agilent). RNA libraries were constructed and sequenced using poly-dT enrichment or ribosomal RNA depletion sample preparation methods in conjunction with DNB sequencing technology on a BGISEQ-500 instrument (BGI). Raw reads were aligned to GRCh38 genome assembly and GENCODE [2] GRCh38.p13 annotation using STAR [3], and then processed with SAMtools [4]. Read counting on genomic features was carried out using Rsubread [5], and the R package DESeq2 [6] was used to perform statistic-based quantification. GENCODE genes with normalised read count of top 60% were considered to be expressed in T-47D cells. DVR calling used RNA-seq and WGS data.

Sequence alignments for RNA-seq and WGS, both in .bam format, were subjected to base quality recalibration procedures described previously using Picard tools [7, 8]. Joint mutation calling was performed using Mutect2 programme in the GATK 3.8 package, with RNA-seq data set as ‘Tumour’ and WGS data set as ‘Normal’. The resultant variant calls went through 14 GATK filters and variant satisfying the “pass” criteria set by Mutect2 were kept for following analysis [9]. The resulting variants were then further subjected to two filters by SNPiR programme[10], removing variants within homopolymer runs and repeat elements, the latter of which were identified by the BLAT algorithm. The resultant variants, together with the RNA-seq alignments were then analysed using the rMATS-DVR programme to identify DVRs [7], where likelihood-ratio test was performed to derive the probability and FDR for the change of alternative allele frequencies (ΔAF) of each allele. Here, AF denotes the alternative allele fraction (AF=(read depth of alternative allele)/(total read depth of allele), and ΔAF denotes fraction change of alternative allele for each DVRs (ΔAF) upon doxycycline induction (ΔAF=AF_dox-treated_-AF_dox-untreated_). To qualify as a DVR, a FDR of ≤0.05 was applied.

## Plasmid cloning

DNA encoding 3×Flag-A3B-GFP were synthesised by GeneArt service (Thermo Fisher Scientific Inc.). Codons were sequence optimised to boost protein expression and distinguish the mRNA of exogenously expressed proteins from their intrinsic counterparts. PCR was used to add an AgeI restriction site and Kozak sequences at the 5’ and a BspD1 restriction site at the 3’-end of the construct. Empty pTRIPZ plasmid was obtained from Horizon Discovery. The DNA fragment was cloned into pTRIPZ plasmid using AgeI and BspD1 restriction digestion and ligation methods with enzymes provided by New England Biolabs. For A3B**-GFP construct, two rounds of site-directed mutagenesis at E68Q and E225Q sites on A3B were performed on 3×Flag-A3B-GFP-containding pTRIPZ plasmid using the Quick-Change kit provided by Agilent. Plasmids encoding the fusion proteins A3A-Cas9n-UGI-NLS, A3Bi-Cas9n-UGI and A3Bi-ctd-Cas9n-UGI-NLS were gifts Reuben Harris and acquired from Addgene.

## Lentivirus packaging

Sequence-verified plasmids were maintained in Mach1 T1 *E. coli* cells. For lentivirus packaging, helper plasmids psPAX2 and pMD2.G were co transfected with pTRIPZ encoding the protein of interest into Lenti-X 293T cells (Clonetech) using calcium-phosphate transfection kit (Promega). After changing the culture medium at 24 hours, virus was harvested at 48 and 72 hours. Virus was pooled and precipitated using Peg-it reagent (System Biosciences) and suspended in serum-free RPMI medium (Gibco). Multiplicity of infection (M.O.I) of viruses were measured using the manufacturer’s guidance (Horizon Discovery).

## eCLIP-seq and data analysis

T-47D cells transduced with lentiviral inducible A3B-GFP were treated with 1 µg/mL doxycycline for 48 hours in petri dishes, followed by aspiration of culture medium and exposure to 254 nm UV as previously described [11] in a Stratalinker instrument. The cells were scraped, frozen, and submitted for eCLIP-sequencing (EclipseBio). Analyses were conducted with two biological repeats, with and anti-flag M2 monoclonal antibody (Sigma) to pull down the A3B-dTAG using the protocol described in ENCODE. In addition, the resultant sequencing read alignment file was subjected to cluster identification using PureCLIP programme [12], and consensus peaks from the two replicates were identified by the MSPC programme [13].

## RNA folding analysis.

RNA folding analysis was conducted using RNAFold [14] programme, with the assistance of R package LncFinder [15]. As input, ±100 bp of SNV or RNA editing sites were subjected to the analysis. For SNVs identified from dbSNP (v150) [16, 17], random sampling (n = 100,000) was applied. For control sites, gene segments with read depth of greater than 10 was first extracted by BEDtools using results from RNA-seq [18], followed by random sampling (n = 1000) of sites matching the interested sequence motif. The resulting folded structure were visually inspected, to give fraction of RNA editing sites in loop structures. For RNAFold analysis, the following additional parameters were used: “-T 37 –salt 1.0 -d 2 --noLP”.

## Sequencing data visualisation

Bigwig files containing enrichment scores across GRCh38 genome assembly was used to plot the heatmaps, profile plots and signal tracks. The R package ‘seqplots’ was used to perform data visualisation with the assistance of a graphical user interface [19]. For heatmaps, average data using a window length of 50 was used, whereas for profile plots, average data with standard deviation (SD) using a window length of 100 was used. Signal profiles across specific genomic regions were visualised by The Integrative Genomics Viewer (IGV) [20].

## RNA immunoprecipitation (RIP)

The Magna RIP kit (Merck) was used to perform RIP, following manufacturer’s instructions. Anti-flag M2 monoclonal antibody and human IgG isotype control (Thermo Fisher) were used to capture protein-bound RNA.

## RNA interference

RNA interference was performed using a method previously described [21]. siRNA oligo targeting A3B (L-017322-00) and ESR1 (L-003401-00) were purchased from Dharmacon. Non-targeting siRNA was purchased from Qiagen (1027281).

## Protein co-immunoprecipitation

T-47D cells were lysed in ice-cold lysis buffer (50 mM HEPES, 150 mM NaCl, 1 mM EDTA, 2.5 mM EGTA, 50 mM NaF, 20 mM β-glycerophosphate, 4 mM Na3VO4, 1 mM DTT and 0.1% Tween-20, pH 8), followed by treatment of benzonase (Sigma) on ice, Protein co-immunoprecipitation was conducted using method previously described [21], with an antibody against intrinsic A3B (Abcam, Ab184990) and IgG isotype control (Thermo Fisher).

## Depletion of NEAT1 and MALAT1

Methods previously describing depletion of lncRNA using ASO locked nucleic acid-gapmeR (Qiagen) were used for transient depletion of NEAT1 and MALAT1 [22, 23]. The sequences for gapmeR oligonucleotides are as follows: 5’-CTCACACGTCCATCT-3’ (NEAT1)；5’- ACATTGCCTCTTCATT-3’ (MALAT1) and 5’- CATACTATATGACAG-3’ (control). For knockdown of NEAT1 and MALAT1, sequences for shRNA targeting both genes were subcloned into pLKO.1 vector (Addgene) as previously described [24, 25], and the vectors were subjected to lentivirus packaging. Cells were transduced with MOI of 100 with the assist of 10 µg/ml polybrene, followed by puromycin (4 µg/mL) selection. Knockdown efficacy was evaluated using RT-qPCR.

## Reverse transcription and quantitative PCR

RT-qPCR were performed with high-capacity cDNA reverse transcription and Power SYBR green kits (Thermo Fisher), and analyses were performed on a ViiA 7 instrument (Thermo Fisher). The following primers were used for NEAT1_1, NEAT1_2 and MALAT1: NEAT1_1-F: 5’-GGCACAAGTTTCACAGGCCTACATGGG-3’, NEAT1_1-R: 5’-GCCAGAGCTGTCCGCCCAGCGAAG-3’; NEAT1_2-F: 5’-GGAGCCAACCTGCCCTGAAT-3’; NEAT1_2-R: 5’-CCACAGGCTACCCTCTGCTC-3’；MALAT1-F：5’-CTTCCCTAGGGGATTTCAGG-3’；MALAT1-R：5’-GCCCACAGGAACAAGTCCTA-3’. For the quantification of A3A, A3B and GREB1, Taqman Gene Expression Assay probes were purchased from ABI with the following references: Hs00377444, Hs00358981 and Hs00536409.

## Measurement of APOBEC3 enzymes’ activity

A method previously described was used to conduct measurement of A3A and A3B activity in live cells using APOBEC-Cas9 system. To quantify for the edited cells expressing eGFP, the Operetta CLS high-content imager was used. Intrinsic A3B activity was quantified in cells using a previously described method. The intrinsic A3B protein was first immunoprecipitated with anti-A3B antibody (Abcam) [21]. The resultant beads were rinsed with deamination assay buffer (50 mM potassium acetate, 20 mM Tris-acetate, 10 mM magnesium acetate, 100 ng/ml BSA, pH 7.9), and subjected to biochemical testing of A3B’s activity using the BspH1 biosensor [26]. To quantify the A3A activity in cells, a method previously described involving measuring DDOST1 C558U editing was used [27].

## Droplet Digital PCR

RNAs were reverse transcribed using the High-Capacity cDNA Reverse Transcription Kit (Thermo Fisher Scientific). Droplet generation was conducted in a QX200 Droplet Generator (Bio-Rad) using manufacturer-provided reagents. Droplets were subjected to PCR amplification in a C1000 Touch Thermal Cycler (Bio-Rad). Post-PCR, droplets were analysed using a QX200 Droplet Reader (Bio-Rad) for FAM and HEX fluorescence, in which gating was based on positive and negative control oligonucleotides. QuantaSoft (Bio-Rad) programme was used for data analysis. Primers for ddPCR experiments was designed and purchased from the web-based design portal hosted by Bio-Rad.

## TCGA data analysis

SNV and normalised RNA-seq data for each BRCA and LUAD patient were downloaded from TCGA and NMF analysis for mutational signature contributions were analysed using R package using ‘TCGAretriever’ and ‘mutSignatures’[28, 29]. For visualisation of analysis results, R package ggplot2 was used[30].

**References**

1 Li H, Durbin R. Fast and accurate short read alignment with Burrows-Wheeler transform. *Bioinformatics* 2009; 25: 1754-1760.

2 Harrow J, Frankish A, Gonzalez JM, Tapanari E, Diekhans M, Kokocinski F *et al*. GENCODE: the reference human genome annotation for The ENCODE Project. *Genome Res* 2012; 22: 1760-1774.

3 Dobin A, Davis CA, Schlesinger F, Drenkow J, Zaleski C, Jha S *et al*. STAR: ultrafast universal RNA-seq aligner. *Bioinformatics* 2013; 29: 15-21.

4 Danecek P, Bonfield JK, Liddle J, Marshall J, Ohan V, Pollard MO *et al*. Twelve years of SAMtools and BCFtools. *Gigascience* 2021; 10.

5 Liao Y, Smyth GK, Shi W. The R package Rsubread is easier, faster, cheaper and better for alignment and quantification of RNA sequencing reads. *Nucleic Acids Res* 2019; 47: e47.

6 Love MI, Huber W, Anders S. Moderated estimation of fold change and dispersion for RNA-seq data with DESeq2. *Genome Biol* 2014; 15: 550.

7 Wang J, Pan Y, Shen S, Lin L, Xing Y. rMATS-DVR: rMATS discovery of differential variants in RNA. *Bioinformatics* 2017; 33: 2216-2217.

8 Neums L, Suenaga S, Beyerlein P, Anders S, Koestler D, Mariani A, Chien J. VaDiR: an integrated approach to Variant Detection in RNA. *Gigascience* 2018; 7: 1-13.

9 Benjamin D, Sato T, Cibulskis K, Getz G, Stewart C, Lichtenstein L. Calling Somatic SNVs and Indels with Mutect2. *bioRxiv* 2019.

10 Piskol R, Ramaswami G, Li JB. Reliable identification of genomic variants from RNA-seq data. *Am J Hum Genet* 2013; 93: 641-651.

11 Van Nostrand EL, Pratt GA, Shishkin AA, Gelboin-Burkhart C, Fang MY, Sundararaman B *et al*. Robust transcriptome-wide discovery of RNA-binding protein binding sites with enhanced CLIP (eCLIP). *Nat Methods* 2016; 13: 508-514.

12 Krakau S, Richard H, Marsico A. PureCLIP: capturing target-specific protein-RNA interaction footprints from single-nucleotide CLIP-seq data. *Genome Biol* 2017; 18: 240.

13 Jalili V, Matteucci M, Masseroli M, Morelli MJ. Using combined evidence from replicates to evaluate ChIP-seq peaks. *Bioinformatics* 2015; 31: 2761-2769.

14 Gruber AR, Lorenz R, Bernhart SH, Neubock R, Hofacker IL. The Vienna RNA websuite. *Nucleic Acids Res* 2008; 36: W70-74.

15 Han S, Liang Y, Ma Q, Xu Y, Zhang Y, Du W *et al*. LncFinder: an integrated platform for long non-coding RNA identification utilizing sequence intrinsic composition, structural information and physicochemical property. *Brief Bioinform* 2019; 20: 2009-2027.

16 Smigielski EM, Sirotkin K, Ward M, Sherry ST. dbSNP: a database of single nucleotide polymorphisms. *Nucleic Acids Res* 2000; 28: 352-355.

17 Sherry ST, Ward M, Sirotkin K. dbSNP-database for single nucleotide polymorphisms and other classes of minor genetic variation. *Genome Res* 1999; 9: 677-679.

18 Quinlan AR, Hall IM. BEDTools: a flexible suite of utilities for comparing genomic features. *Bioinformatics* 2010; 26: 841-842.

19 Stempor P, Ahringer J. SeqPlots - Interactive software for exploratory data analyses, pattern discovery and visualization in genomics. *Wellcome Open Res* 2016; 1: 14.

20 Robinson JT, Thorvaldsdottir H, Turner D, Mesirov JP. igv.js: an embeddable JavaScript implementation of the Integrative Genomics Viewer (IGV). *Bioinformatics* 2023; 39.

21 Zhang C, Stockwell SR, Elbanna M, Ketteler R, Freeman J, Al-Lazikani B *et al*. Signalling involving MET and FAK supports cell division independent of the activity of the cell cycle-regulating CDK4/6 kinases. *Oncogene* 2019; 38: 5905-5920.

22 Adriaens C, Standaert L, Barra J, Latil M, Verfaillie A, Kalev P *et al*. p53 induces formation of NEAT1 lncRNA-containing paraspeckles that modulate replication stress response and chemosensitivity. *Nat Med* 2016; 22: 861-868.

23 Amodio N, Stamato MA, Juli G, Morelli E, Fulciniti M, Manzoni M *et al*. Drugging the lncRNA MALAT1 via LNA gapmeR ASO inhibits gene expression of proteasome subunits and triggers anti-multiple myeloma activity. *Leukemia* 2018; 32: 1948-1957.

24 Qu D, Sun WW, Li L, Ma L, Sun L, Jin X *et al*. Long noncoding RNA MALAT1 releases epigenetic silencing of HIV-1 replication by displacing the polycomb repressive complex 2 from binding to the LTR promoter. *Nucleic Acids Res* 2019; 47: 3013-3027.

25 Zhang P, Cao L, Zhou R, Yang X, Wu M. The lncRNA Neat1 promotes activation of inflammasomes in macrophages. *Nat Commun* 2019; 10: 1495.

26 Zhang YH, Guo XC, Zhong JB, Zhong DX, Huang XH, Fang ZY *et al*. Discovery of APOBEC Cytidine Deaminases Inhibitors Using a BspH1 Restriction Enzyme-Based Biosensor. *Chemistryselect* 2022; 7.

27 Jalili P, Bowen D, Langenbucher A, Park S, Aguirre K, Corcoran RB *et al*. Quantification of ongoing APOBEC3A activity in tumor cells by monitoring RNA editing at hotspots. *Nat Commun* 2020; 11: 2971.

28 Fantini D, Vidimar V, Yu Y, Condello S, Meeks JJ. MutSignatures: an R package for extraction and analysis of cancer mutational signatures. *Sci Rep* 2020; 10: 18217.

29 Cancer Genome Atlas Research N, Weinstein JN, Collisson EA, Mills GB, Shaw KR, Ozenberger BA *et al*. The Cancer Genome Atlas Pan-Cancer analysis project. *Nat Genet* 2013; 45: 1113-1120.

30 Wickham H. ggplot2 : Elegant Graphics for Data Analysis. *Use R!,*, 2nd edn. Springer International Publishing : Imprint: Springer,: Cham, 2016, pp 1 online resource (XVI, 260 pages 232 illustrations, 140 illustrations in color.

31 Consortium EP. The ENCODE (ENCyclopedia Of DNA Elements) Project. *Science* 2004; 306: 636-640.

# Supplemental Tables

| Type of DVR | Total # of RVs detected | | % RVs overlapping WGS SNV | | Total # of DVRs detected | | % DVRs overlapping WGS SNV | | % DVRs overlapping dbSNP SNV | |
| --- | --- | --- | --- | --- | --- | --- | --- | --- | --- | --- |
|  | rMATS-DVR | This study | rMATS-DVR | This study | rMATS-DVR | This study | rMATS-DVR | This study | rMATS-DVR | This study |
| A-C | 3002 | 3 | 76.32 | 33.33 | 127 | 0 | 59.84 | 0 | 48.82 | 0 |
| ***A-G(I)*** | ***86726*** | ***3683*** | ***12.67*** | ***0.03*** | ***7623*** | ***272*** | ***3.65*** | ***0*** | ***10.40*** | ***9.19*** |
| A-U | 2182 | 14 | 71.95 | 0 | 119 | 1 | 59.66 | 0 | 58.82 | 100.00 |
| C-A | 3143 | 21 | 79.29 | 4.76 | 144 | 3 | 60.42 | 0 | 44.44 | 0 |
| C-G | 4100 | 4 | 84.46 | 0 | 180 | 2 | 65.00 | 0 | 47.78 | 0 |
| ***C-U*** | ***14205*** | ***1093*** | ***81.44*** | ***0*** | ***930*** | ***511*** | ***36.02*** | ***0*** | ***40.75*** | ***18.98*** |
| G-A | 14268 | 667 | 84.62 | 0 | 557 | 13 | 71.63 | 0 | 57.99 | 7.69 |
| G-C | 4082 | 7 | 82.73 | 0 | 165 | 1 | 62.42 | 0 | 45.45 | 0 |
| G-U | 3469 | 17 | 78.32 | 0 | 151 | 2 | 60.93 | 0 | 51.66 | 0 |
| U-A | 2008 | 5 | 75.15 | 0 | 84 | 1 | 54.76 | 0 | 48.81 | 0 |
| U-C | 13521 | 348 | 75.97 | 0.57 | 388 | 2 | 69.07 | 0 | 67.53 | 50.00 |
| U-G | 2829 | 14 | 83.14 | 0 | 123 | 0 | 72.36 | 0 | 55.28 | 0 |

**Supplemental Table 1:** Comparison of results for various types of RNA variants (RVs) and DVRs identified by the original rMATS-DVR method and the method used in this study. Samples prepared using poly-A enrichment, comparing T_72_ and T_0_. SNVs from T-47D cells were derived from data curated in GSE193225.

| Type of DVR | Total # of RVs detected | | % RVs overlapping WGS SNV | | Total # of DVRs detected | | % DVRs overlapping WGS SNV | | % DVRs overlapping dbSNP SNV | |
| --- | --- | --- | --- | --- | --- | --- | --- | --- | --- | --- |
|  | rMATS-DVR | This study | rMATS-DVR | This study | rMATS-DVR | This study | rMATS-DVR | This study | rMATS-DVR | This study |
| A-C | 10175 | 8 | 78.58 | 0 | 203 | 0 | 60.59 | 0 | 53.20 | 0 |
| ***A-G(I)*** | ***303009*** | ***27958*** | ***13.23*** | ***0.04*** | ***26255*** | ***1451*** | ***1.90*** | ***0*** | ***9.43*** | ***9.10*** |
| A-U | 7718 | 26 | 78.35 | 7.69 | 150 | 2 | 69.33 | 0 | 47.33 | 50.00 |
| C-A | 10459 | 28 | 81.91 | 3.57 | 225 | 5 | 73.33 | 0 | 48.44 | 0 |
| C-G | 14024 | 11 | 84.15 | 9.09 | 254 | 3 | 67.72 | 0 | 58.66 | 0 |
| ***C-U*** | ***46797*** | ***1852*** | ***85.88*** | ***0*** | ***1540*** | ***739*** | ***38.12*** | ***0*** | ***40.13*** | ***14.61*** |
| G-A | 48037 | 409 | 88.78 | 0 | 811 | 8 | 76.82 | 0 | 67.45 | 25.00 |
| G-C | 14360 | 21 | 79.60 | 0 | 291 | 2 | 62.20 | 0 | 51.20 | 0 |
| G-U | 11974 | 26 | 81.85 | 0 | 250 | 4 | 65.20 | 0 | 50.00 | 0 |
| U-A | 7619 | 24 | 76.53 | 0 | 170 | 0 | 54.12 | 0 | 54.71 | 0 |
| U-C | 45876 | 1171 | 79.34 | 0.26 | 652 | 2 | 80.67 | 0 | 66.26 | 50.00 |
| U-G | 10995 | 16 | 84.03 | 6.25 | 224 | 1 | 64.73 | 0 | 57.59 | 0 |

**Supplemental Table 2:** Comparison of results for various types of RNA variants (RVs) and DVRs identified by rMATS-DVR and the method used in this study. Samples prepared using ribosomal depletion, comparing T_72_ and T_0_. SNVs from T-47D cells were derived from data curated in GSE193225.

# Supplemental Lists

**Supplementary List 1:** List of DVRs detected in this study.

**Supplementary List 2:** Results for differential gene expression analysis using RNA-seq data.

# Supplemental Figures

**Supplemental Figure 1: Identification of A3B-mediated DVRs using a lentiviral inducible system.** (A). Quantitative RT-PCR showing the transcript level of endogenous (blue) and inducible-exogenous (red) A3B following induction using 100 ng/ml doxycycline. Data represents mean of n=3 biological replicates and error bars for standard deviation (SD). **** and ns denotes p<0.0001 and non-significance respectively by Student’s T test. (B). Table showing number of variants identified in various stages of the analysis pipeline. (C). Dot plot showing the fraction change of alternative allele (ΔAF) of DVRs common in both 72- and 24-hour induced T-47D cells. The dotted line shows y = x.

**Supplemental Figure 2: Identification of DVRs attributable to the effects of doxycycline.** (A). Volcano plot depicting fraction change of alternative allele for each DVRs (ΔAF) upon doxycycline treatment against false discovery rate (FDR) derived from likelihood-ratio test. Data were derived from two groups of n = 4 independent repeats comparing the effect of DMSO versus doxycycline (1 μg/ml) following 72 hours exposure in T-47D cells. (B). Table showing break-down of DVRs identified in (A).

**Supplemental Figure 3: Quantification of transcript levels for APOBEC and ADAR family deaminases upon ectopic induction of A3B.** Column graph showing transcript levels for APOBEC (A) and ADAR (B) deaminases following induction of A3B-GFP. Data represent average of n = 4 experiments using normalised RNA-seq read counts by transcript per million (TPM) method, and error bars represent standard deviation. * and **** denotes FDR < 0.05 and < 0.0001 using DESeq2 statistics. (C). Immunoblotting of ADAR and ADARB2 in samples with induced A3B-GFP or A3B**-GFP expression.

**Supplemental Figure 4: Representative snapshots from the Integrated Genome Viewer (IGV) showing level of base editing at identified DVRs (labelled with arrow).**

**Supplemental Figure 5: Predicted folded structures for RNA harbouring selected C>U DVRs.** Structure predicted using RNAFold hosted by ViennaRNA services.

**Supplemental Figure 6: Frequency of sequence motifs at A3B-binding RNA clusters identified by eCLIP-seq.** (A). Graph depicting probability of indicated sequence motif across sequences flanking A3B crosslinking sites predicted by PureCLIP and MSPC using eCLIP-seq results. (B). Logo for the motif used in (A), representing sequence motif at A3B-mediated C>U DVRs.

**Supplemental Figure 7:** **Differential gene expression analysis for RNA samples from T-47D cells expressing inducible A3B or A3B**.** Volcano plots of false discovery rates versus fold change in expression for differentially expressed genes (DEGs), where DEGs with A>G(I) DVRs are coloured in blue and DEGs with C>U DVRs in red. For each type of DVRs a 2 Χ 2 contingency table analysis was performed and the p value denotes results from Χ^2^ test.

**Supplemental Figure 8: Comparative analysis of C>U editing levels at DVRs identified in cells expressing A3B-GFP versus A3B**-GFP.** Dot plot comparing the fraction change of alternative allele (ΔAF) of variants identified in A3B-GFP and A3B**-GFP T-47D cells following 72-hour induction by doxycycline. Data denote average of n = 4 independent experiments.

**Supplemental Figure 9: Binding of A3B at *NEAT1* and *MALAT1* loci.** Signal tracks for A3B eCLIP-seq at *NEAT1* (A) and *MALAT1* (B) loci. The signal was normalised using eCLIP data standards described by ENCODE [31]. Also shown are locations for C>U DVRs and A3B binding clusters identified by PureCLIP.

**Supplemental Figure 10: Investigating the influence of estrogen receptor signalling on A3B-mediated C>U RNA editing.** (A). Immunoblot analysis demonstrating effective knockdown of ESR1 using siRNA in T-47D cells. (B). Normalised transcript levels of A3B, NEAT1, and MALAT1 after 72 hours treatment of T-47D cells with ESR1-targeting siRNA, compared to non-targeting (NT) siRNA controls. (C). C>U editing of NEAT1 and MALAT1, quantified by ddPCR following 72 hours of indicated siRNA treatment in T-47D cells. (D). Results from RIP-qPCR with co-immunoprecipitation of over-expressed flag-tagged A3B in T-47D cells cultured with charcoal-stripped foetal bovine serum, treated with DMSO or 100 nM estradiol for 12 hours. Immunoblots confirming successful immunoprecipitation are displayed. (E). Transcript levels of GREB1, NEAT1, and MALAT1 measured by qPCR in T-47D cells treated with DMSO or 100 nM estradiol for 12 hours. For panels (B)-(E), data represent the mean ± SD of three biological replicates. Statistical significance was assessed using two-tailed Student’s T-test, with *, ***, and n.s. indicating p < 0.05, p < 0.001, and non-significant, respectively.

**Supplemental Figure 11: Validation of knockdown efficiency for ASOs and shRNAs against NEAT1 and MALAT1.** Quantification of transcript levels for (A). NEAT1 and MALAT1 or (B). A3A and A3B following ASO transfection in 293T cells. Quantification of transcript levels for (C). NEAT1 and MALAT1 or (D). A3A and A3B following stable expression of shRNAs by lentiviral transduction. For (A)-(D), data represent n=3 biological repeats. Data were normalised against negative control ASO or scramble shRNA. *, **, and *** denote p<0.05, 0.01 and 0.001 using two-tailed Student’s T test.

**Supplemental Figure 12: Expression of A3A, A3B, NEAT1, and MALAT1 in patient data curated from the TCGA database.** (A)-(B). Heatmaps displaying the correlation between the transcript levels of the indicated genes in breast cancer (BRCA) and lung adenocarcinoma patients (LUAD). (C)-(D). Scatter plots illustrating the normalized expression levels of A3A, A3B, NEAT1, and MALAT1 across patient samples. Data points for patients whose gene expression falls within the highest tercile are highlighted in red.

**Supplemental Figure 13: NEAT1 and MALAT1 expression level associates with APOBEC mutational signatures in cancer patients.** Scatter plots showing degree of genomic DNA mutations attributed to APOBEC activity, as measured by sum of SBS2 and SBS13, in breast cancer (A) and lung adenocarcinoma (B) curated in TCGA database. For grouping of patients, normalised RNA-seq data was extracted for NEAT1, MALAT1, A3A and A3B mRNA curated in the TCGA database. * and *** denote p<0.05, and 0.001 using one-way ANOVA.
