# Supplementary figures and images for: Characterisation of APOBEC3B-Mediated RNA editing in breast cancer cells reveals regulatory roles of NEAT1 and MALAT1 lncRNAs
Source: Oncogene. 2024 Sep 25;43(46):3366–77. doi: 10.1038/s41388-024-03171-5 (PMC11554567; doi:10.1038/s41388-024-03171-5)

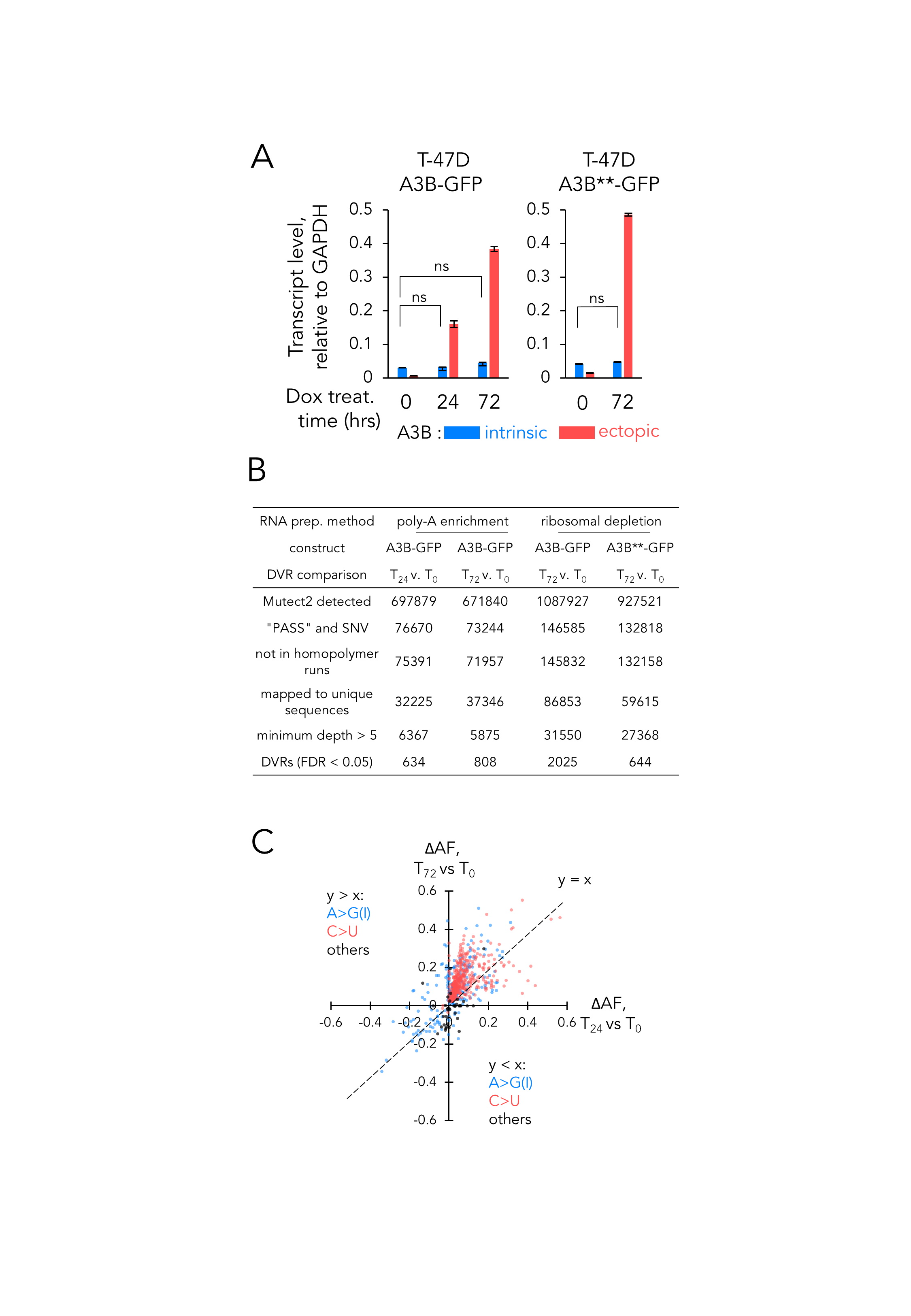

Supplement: Supplementary file 2 — Supplemental Figure 1 [file 41388_2024_3171_MOESM2_ESM.tif]

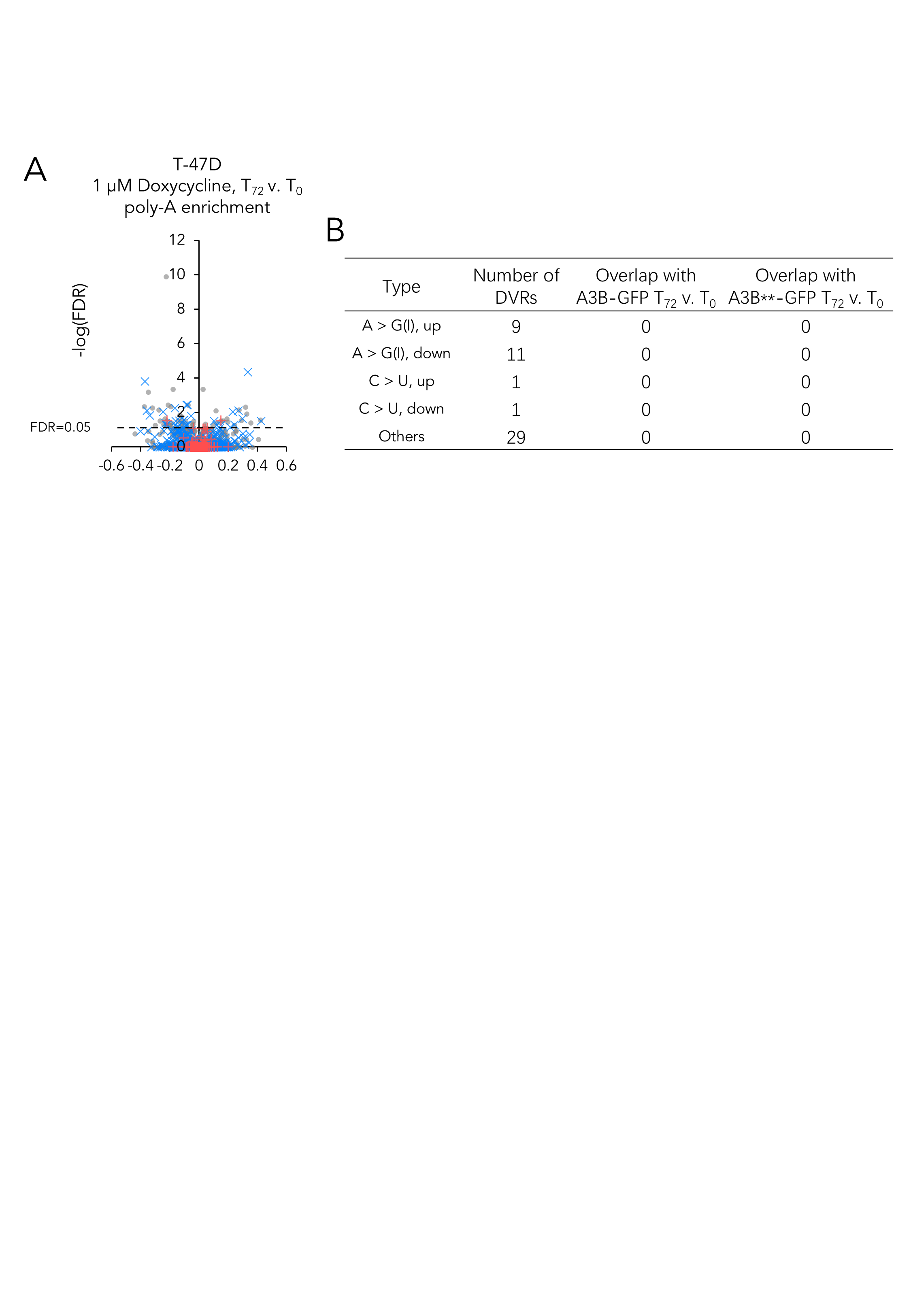

Supplement: Supplementary file 3 — Supplemental Figure 2 [file 41388_2024_3171_MOESM3_ESM.tif]

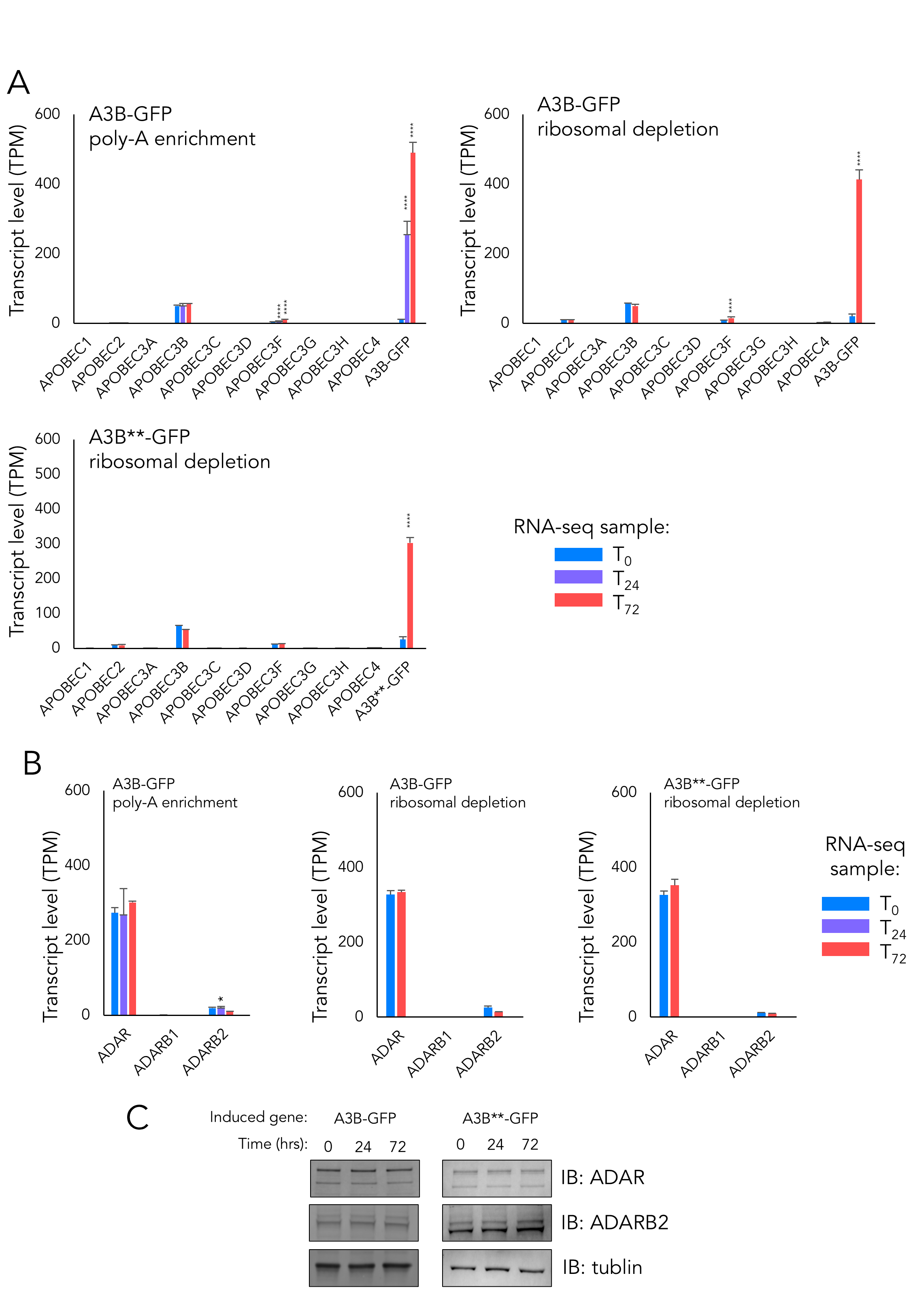

Supplement: Supplementary file 4 — Supplemental Figure 3 [file 41388_2024_3171_MOESM4_ESM.tif]

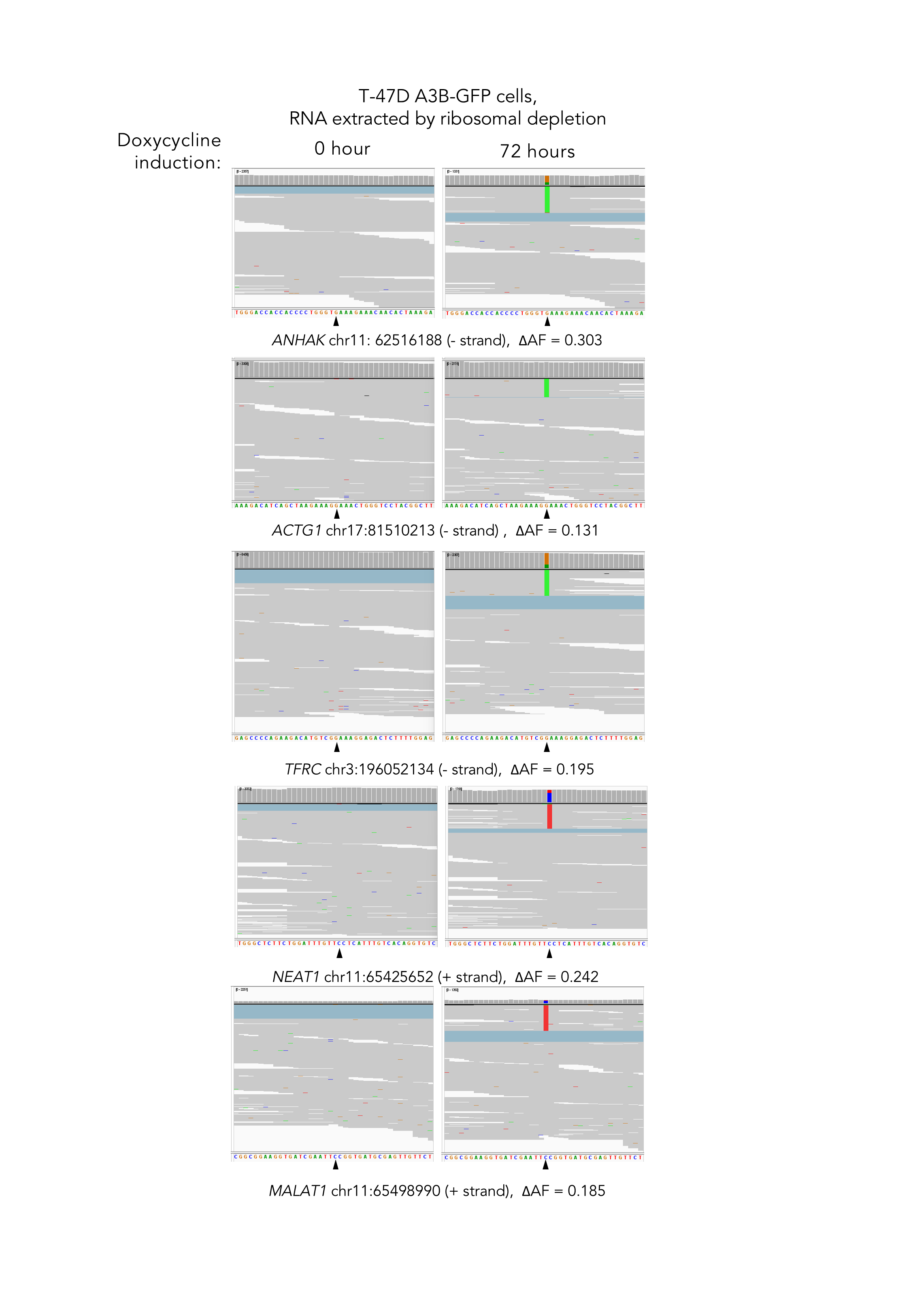

Supplement: Supplementary file 5 — Supplemental Figure 4 [file 41388_2024_3171_MOESM5_ESM.tif]

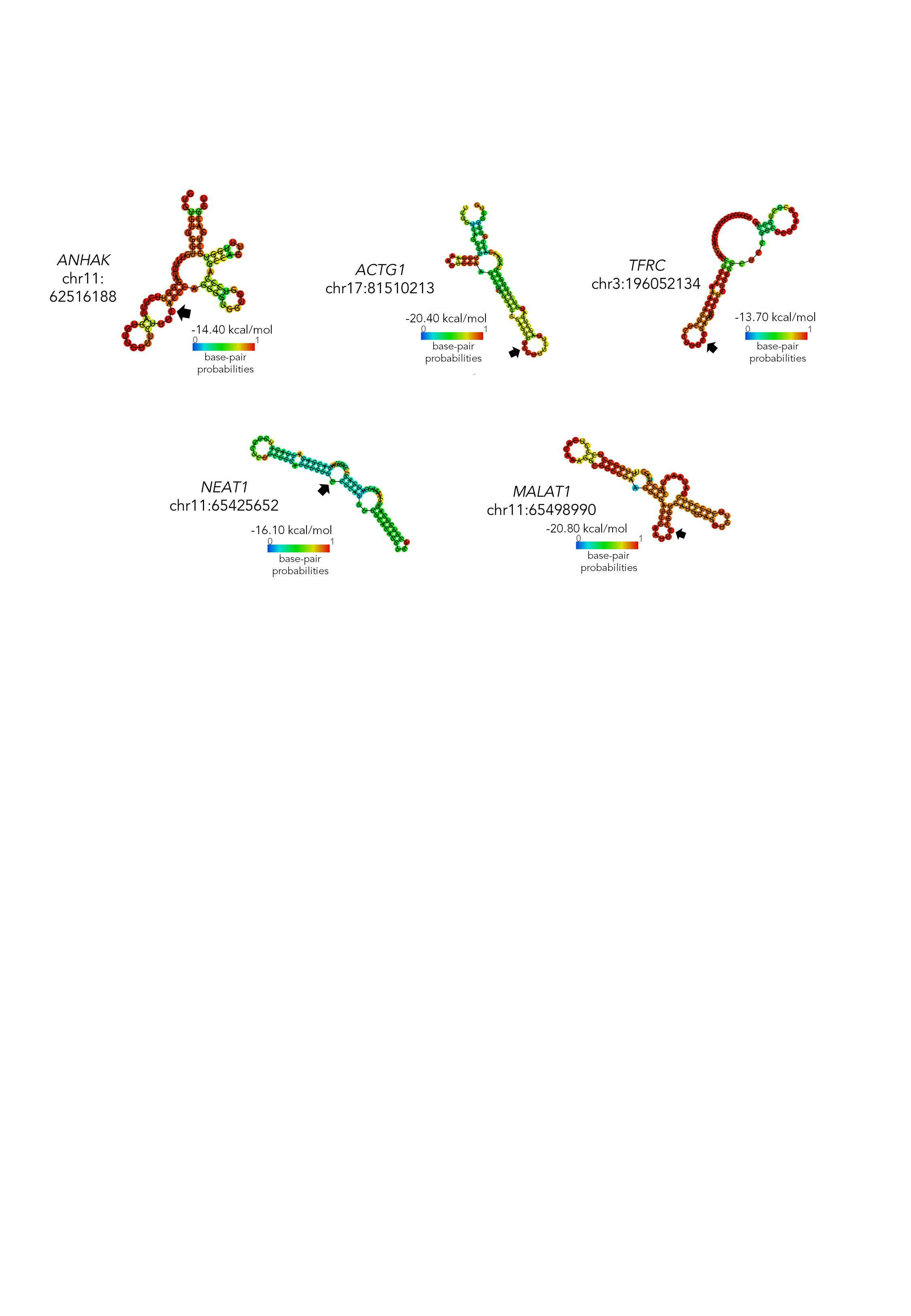

Supplement: Supplementary file 6 — Supplemental Figure 5 [file 41388_2024_3171_MOESM6_ESM.tif]

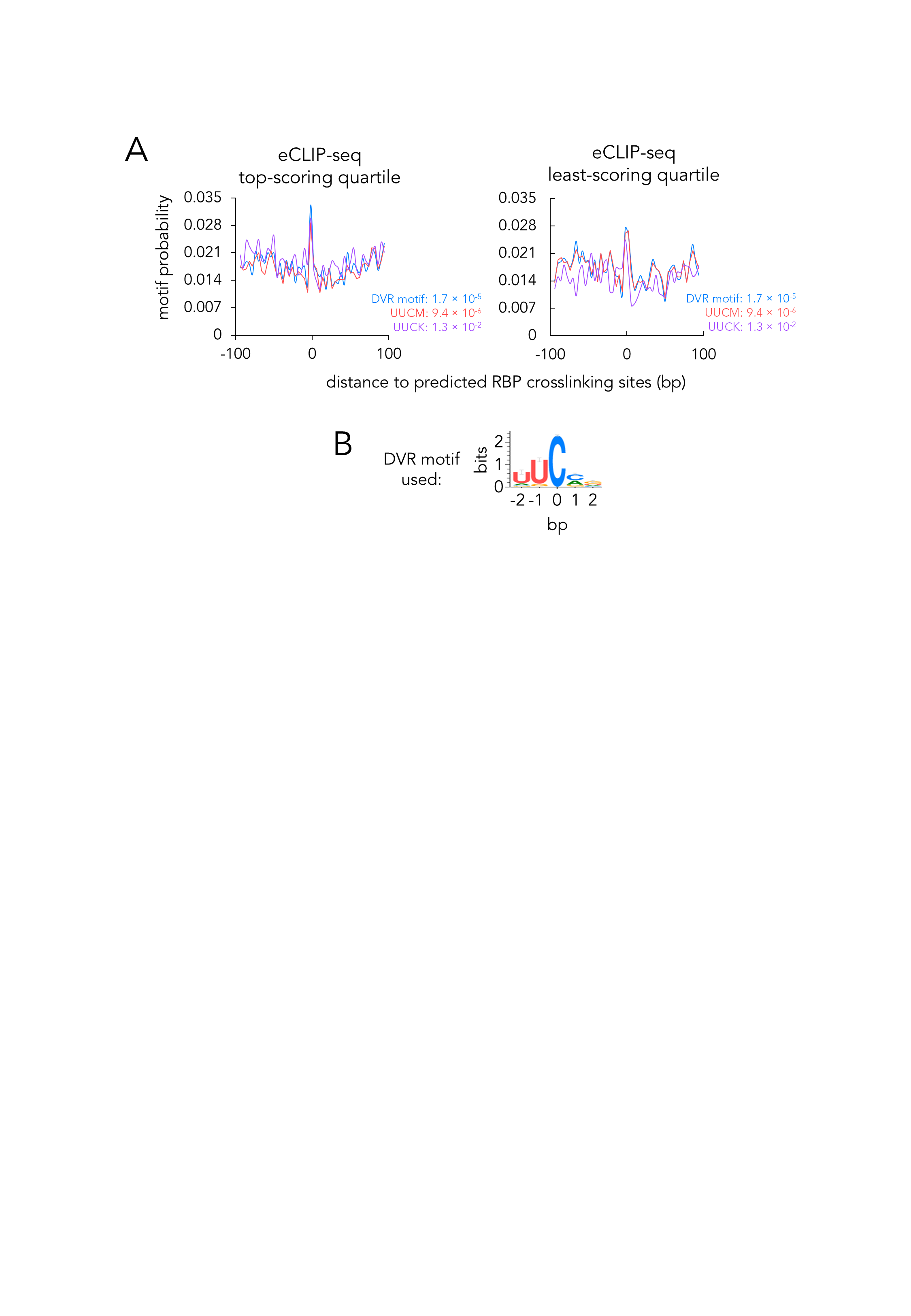

Supplement: Supplementary file 7 — Supplemental Figure 6 [file 41388_2024_3171_MOESM7_ESM.tif]

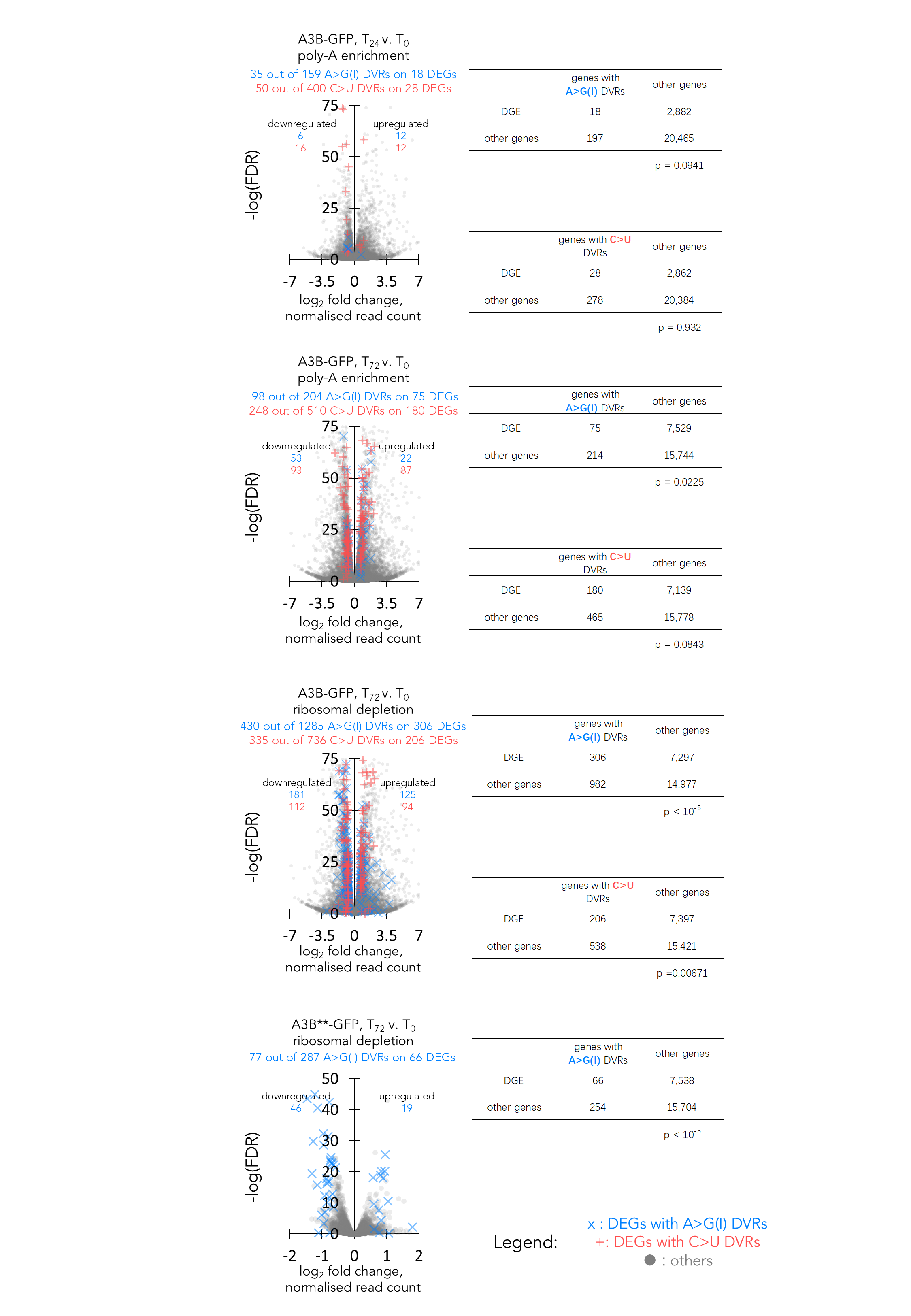

Supplement: Supplementary file 8 — Supplemental Figure 7 [file 41388_2024_3171_MOESM8_ESM.tif]

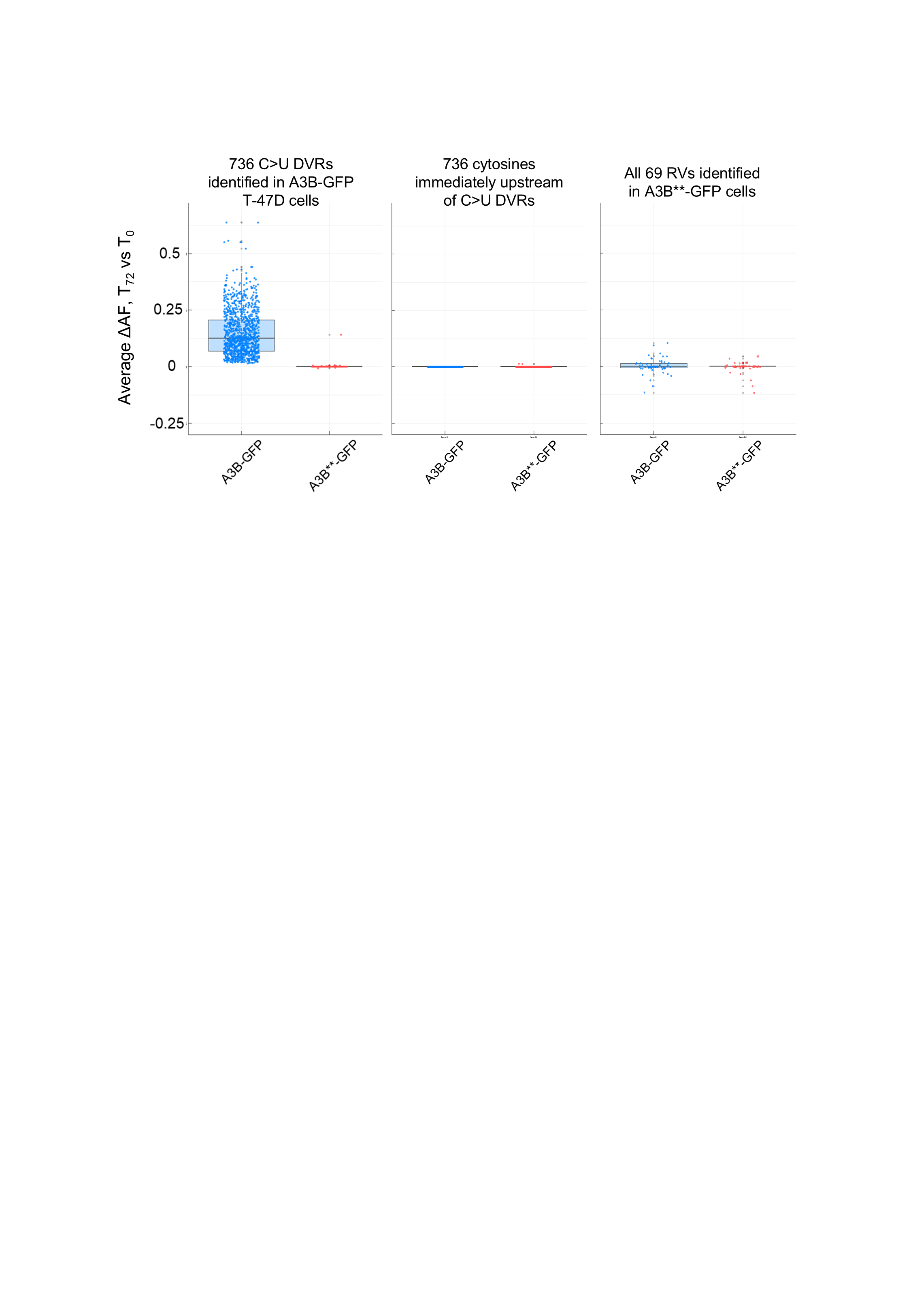

Supplement: Supplementary file 9 — Supplemental Figure 8 [file 41388_2024_3171_MOESM9_ESM.tif]

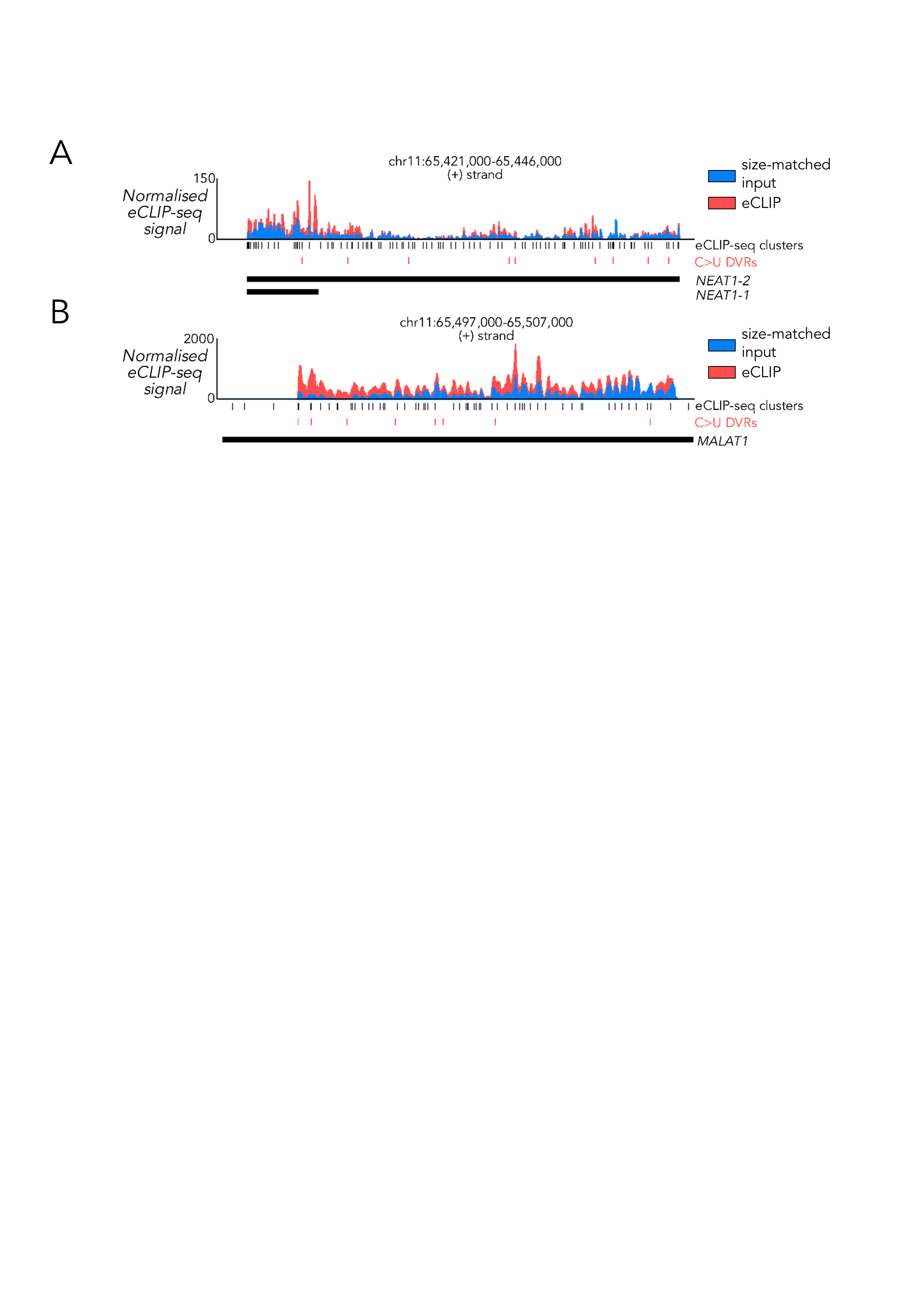

Supplement: Supplementary file 10 — Supplemental Figure 9 [file 41388_2024_3171_MOESM10_ESM.tif]

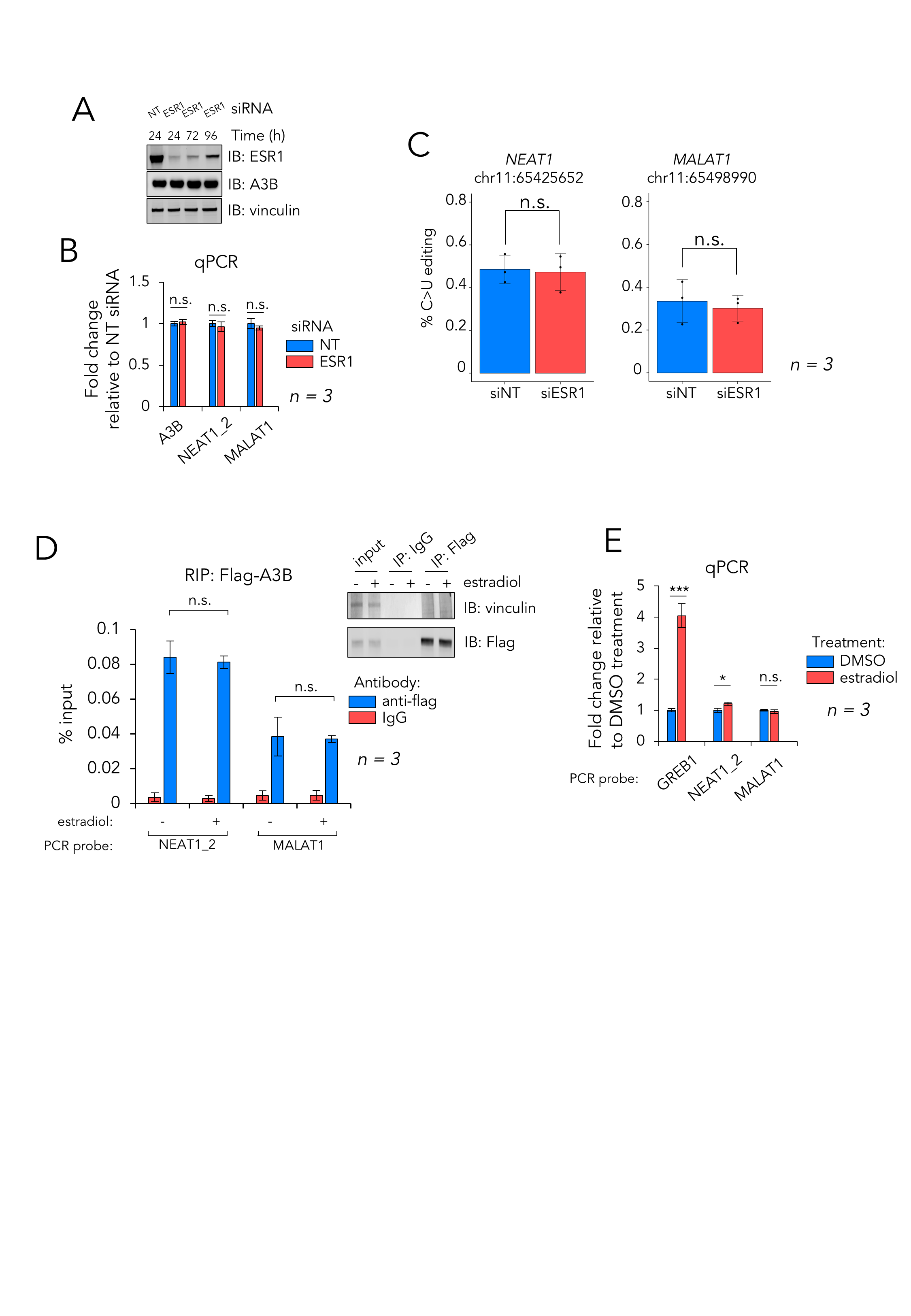

Supplement: Supplementary file 11 — Supplemental Figure 10 [file 41388_2024_3171_MOESM11_ESM.tif]

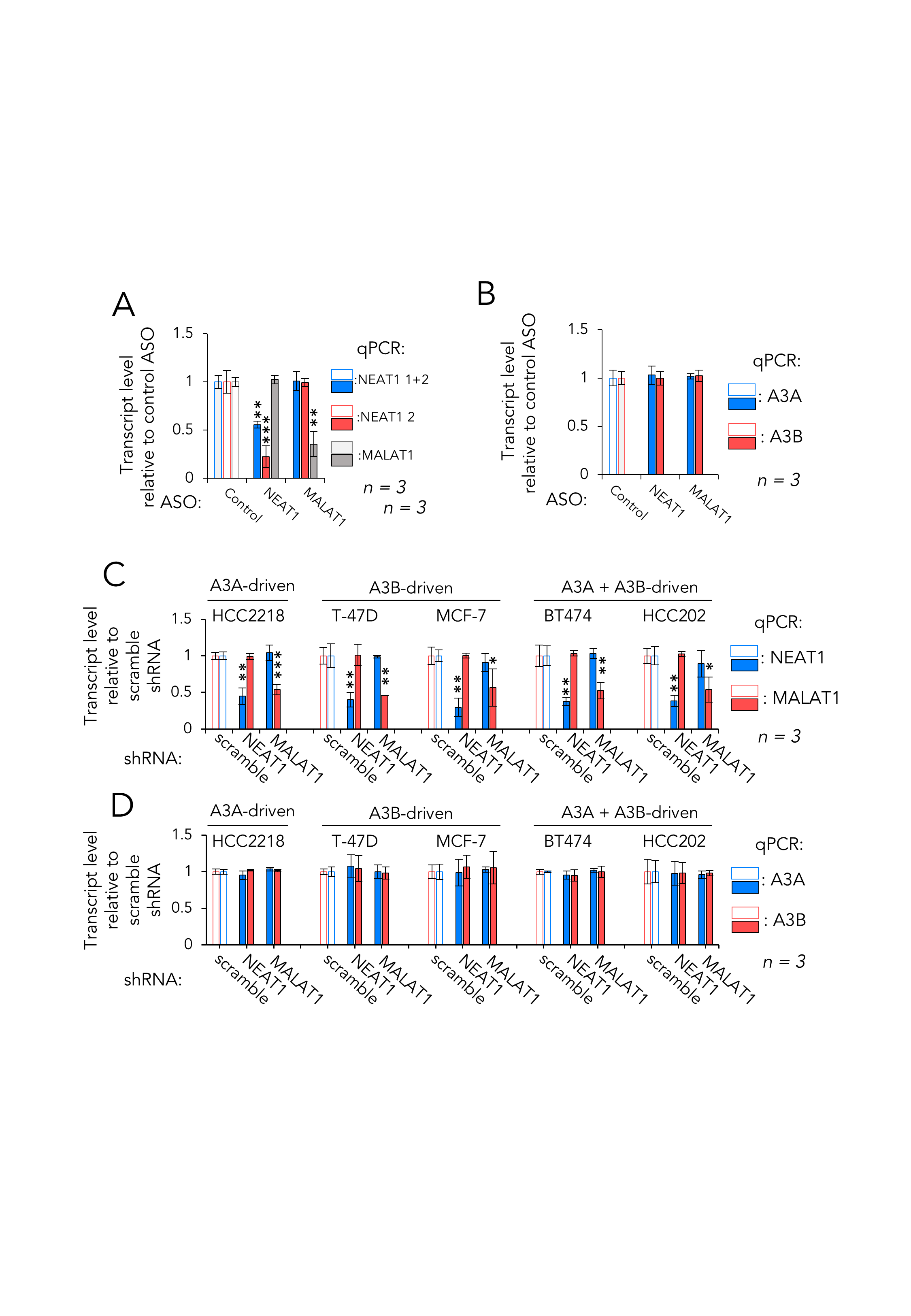

Supplement: Supplementary file 12 — Supplemental Figure 11 [file 41388_2024_3171_MOESM12_ESM.tif]

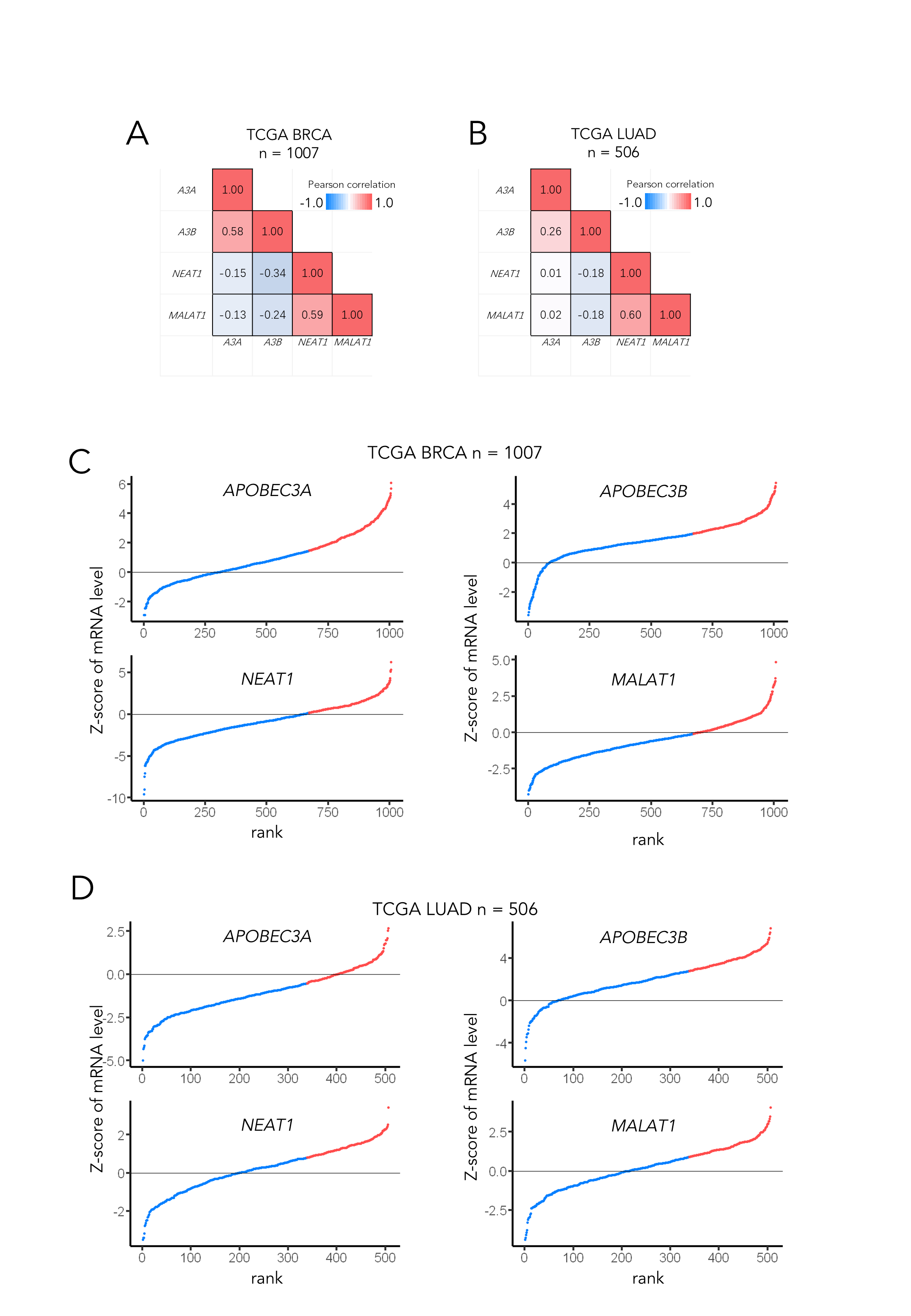

Supplement: Supplementary file 13 — Supplemental Figure 12 [file 41388_2024_3171_MOESM13_ESM.tif]

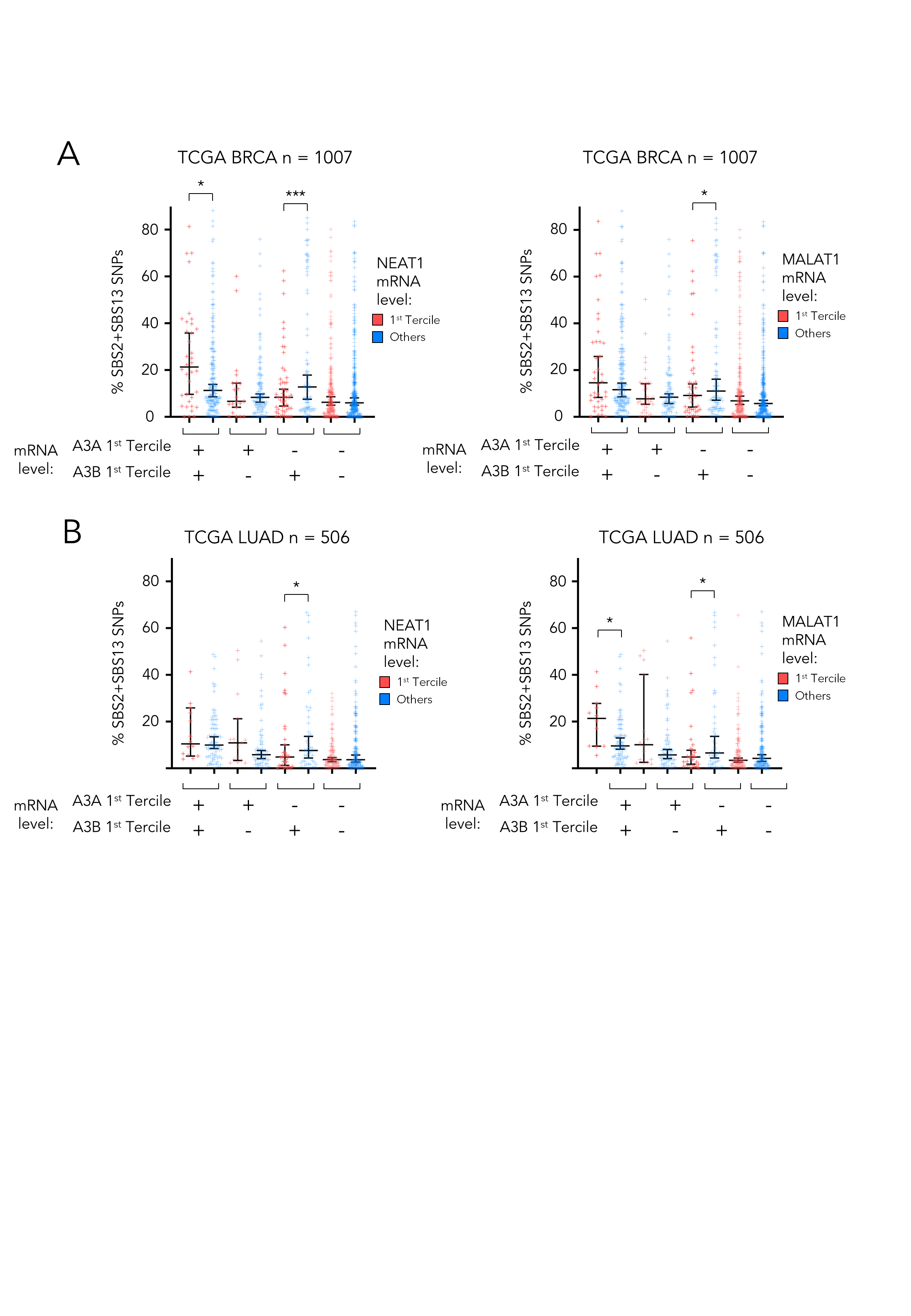

Supplement: Supplementary file 14 — Supplemental Figure 13 [file 41388_2024_3171_MOESM14_ESM.tif]
